# Presbyopia-Correcting Intraocular Lenses Implantation in Eyes After Corneal Refractive Laser Surgery: A Meta-Analysis and Systematic Review

**DOI:** 10.3389/fmed.2022.834805

**Published:** 2022-04-11

**Authors:** Yang Sun, Yingying Hong, Xianfang Rong, Yinghong Ji

**Affiliations:** ^1^Department of Ophthalmology, Eye Institute, Eye and ENT Hospital, Fudan University, Shanghai, China; ^2^National Health Commission (NHC) Key Laboratory of Myopia, Fudan University, Shanghai, China; ^3^Key Laboratory of Myopia, Chinese Academy of Medical Sciences, Shanghai, China; ^4^Shanghai Key Laboratory of Visual Impairment and Restoration, Shanghai, China

**Keywords:** corneal refractive surgery, presbyopia-correcting intraocular lenses, meta-analysis, systematic review, efficacy and safety

## Abstract

**Purpose:**

To assess the efficacy, safety, and predictability of presbyopia-correcting intraocular lenses (IOLs) in cataract patients with previous corneal refractive surgery.

**Methods:**

A systematic literature search was performed to identify studies evaluating the clinical outcomes of presbyopia-correcting IOLs implantation in cataract surgery after laser refractive surgery. Outcomes were efficacy, safety and predictability parameters.

**Results:**

The authors identified 13 studies, involving a total of 128 patients and 445 eyes. Presbyopia-correcting IOLs were effective at improving distance, intermediate and near visual acuity aftercataract surgery. The proportion of post-laser surgery eyes with uncorrected distance visual acuity (UDVA) ≥ 20/25 was 0.82 [95% confidence interval (CI), 0.74-0.90] and the pooled rates of spectacle independence at near, intermediate, and far distances were 0.98 (95% CI, 0.94-1.00), 0.99 (95% CI, 0.95-1.00) and 0.78 (95% CI, 0.65-0.94) respectively. The percentage of participants who suffered from halos and glare was 0.40 (95% CI, 0.25-0.64) and 0.31 (95% CI, 0.16-0.60), respectively. The predictability had a percentage of 0.66 (95% CI, 0.57-0.75) and 0.90 (95% CI, 0.85-0.96) of eyes within ±0.5 diopters (D) and ±1.0 D from the targeted spherical equivalent.

**Conclusions:**

Presbyopia-correcting IOLs provide satisfactory results in terms of efficacy, safety and predictability in patients with previous corneal refractive surgery, but have a higher risk of photopic side effects such as halos and glare.

## Introduction

Laser refractive surgery, considered as the mainstay of refractive surgery, has been well established as a safe and effective treatment for refractive error (1, 2). The corneal refractive surgery procedures that are most commonly performed include photorefractive keratectomy (PRK), laser *in-situ* keratomileusis (LASIK) and small incision lenticule extraction (SMILE). Currently, LASIK is widely accepted all over the world with high quality of life and patient satisfaction (3). With time, post-refractive surgery patients develop symptomatic cataract, possibly presenting earlier than those without history of previous corneal refractive surgery (4). Accustomed to being spectacle independent after corneal refractive surgery, they might seek to remain spectacle-free after cataract surgery (5). One option would be the use of presbyopia-correcting intraocular lenses (IOLs). Presbyopia-correcting IOLs are generally classified into three main categories: multifocal IOLs (MIOLs) including diffractive or refractive designs, extended depth-of-focus (EDOF) IOLs, and accommodative IOLs (6). It has been shown that presbyopia-correcting IOLs can successfully restore both near and intermediate vision together with high spectacle independence compared to monofocal IOLs. However, presbyopia-correcting IOLs are frequently associated with side effects, such as photic disturbances, a decrease in contrast sensitivity, and a high incidence of residual refractive errors, which have an impact on patient satisfaction (6).

Currently, there are only a few studies investigating the visual outcomes of presbyopia-correcting IOLs implantation in cataract surgery after corneal refractive surgery. Thus, we conducted a systematic review of the existing studies on presbyopia-correcting IOLs implantation in post corneal refractive surgery, in respect of the uncorrected and corrected monocular visual acuity at near and distance, the refractive outcomes, the rate of spectacle independence as well as the photic phenomena such as halos and glare.

The purpose of the current study is to summarize and evaluate the evidence regarding the efficacy, safety and predictability of various options of presbyopia-correcting IOLs in cataract patients with previous corneal refractive surgery and to make recommendations on the management based on current clinical knowledge.

## Methods

### Study Inclusion and Exclusion Criteria

Eligible for inclusion were clinical studies published in full text or abstract, evaluating the clinical outcomes of presbyopia-correcting IOLs implantation in cataract surgery after laser refractive surgery.

Studies were included in this study if they were confirmed to meet the following inclusion criteria: (1) population: patients who had corneal refractive laser surgery and subsequent cataract surgery or refractive lenses exchange, (2) intervention: presbyopia-correcting IOL implantation, (3) study design: observational studies, prospective or retrospective, randomized controlled trial (RCT), controlled, or case series, (4) studies reported data on at least one of the following outcome measurement: efficacy, safety and predictability. Exclusion criteria included (1) studies on analysis of IOL power calculation methods, (2) eyes that have not in-the-bag fixed IOL implantation.

### Method of Literature Search

A literature search was performed in Pubmed MEDLINE, Ovid MEDLINE, Web of science, EBM Reviews (all Cochrane Library), Scopus—Health Sciences, ISI Web of Knowledge, EBSCO (Academic Search Complete, CINAHL and ERIC). A manual search of the reference lists of included articles and relevant systematic reviews was conducted to locate additional studies. We used Boolean logic operator through a combination of MeSHs, Entry Terms and Keywords to identify studies. Complete search strategies for each database were described in Supplementary Table S1. There was no restriction on publication year, study design, or language.

All titles and abstracts of papers identified by the search strategies were screened independently by two researchers (Y.S. and Y.H.), and disagreements were resolved through discussions and consultations with a third investigator (X.R.). The reference software EndNote (version X9, Philadelphia, PA, USA) was used to manage the retrieved records and remove the duplicate records. Full texts of all potentially eligible articles were retrieved for detailed assessment according to predetermined criteria.

### Outcomes Measures

The following visual outcomes were documented at the last follow-up time: uncorrected distance visual acuity (UDVA), corrected distance visual acuity (CDVA), uncorrected intermediate visual acuity (UIVA) and uncorrected near visual acuity (UNVA). We only extracted logMAR visual acuity for meta-analysis. Efficacy was interpreted as the proportion of post-laser surgery eyes with a postoperative UDVA ≥ 20/25 and the reported rates of spectacle independence at near, intermediate, and far distances. The safety factors analyzed consisted of the photopic side effects such as halos and glare. The refractive results were evaluated in terms of the postoperative spherical equivalent and the predictability (percentage of eyes within ±0.5D and ±1.0D from the targeted spherical equivalent).

### Data Extraction

Data were extracted by two of the authors (Y.S. and Y.H.) independently and combined by a third reviewer (X.R.). If the data and the methods for obtaining it were considered relatively homogeneous, a meta-analysis was conducted. For continuous data like visual acuity, the mean values and standard deviations were extracted. For categorical data, the number of events were extracted.

### Quality Assessment

Each included study was assigned a level of evidence based on the Oxford Centre for Evidence-based Medicine (CEBM) and adopted by the American Academy of Ophthalmology (7). Methodological Index for Non-randomized studies (MINORS) have been used to assess the quality of non-randomized studies (8). The checklist for included studies uses eight criteria for non-comparative studies and four additional criteria in the case of comparative studies. Each component was scored 0 (not reported), 1 (reported but inadequate), or 2 (reported and adequate). The ideal score for non-comparative studies is 16 and for comparative studies is 24. With a score of 0-8 or 0-12, the risk of bias classification was low, 9-12 or 13-18 was considered fair quality, and 13-16 or 19-24 was high risk of bias for non-comparative and comparative studies, respectively. Two investigators (Y.S. and Y.H.) evaluated the quality of each study independently, with disagreements resolved through discussion and consensus.

### Statistical Analysis

The statistical analysis was carried out using the meta-package in *R* language (version 3.6.0). Statistical heterogeneity between studies was tested by means of a chi-square statistics with an I^2^ value exceeding 50% and a *P*-value of <0.05 of statistical significance. In the absence of statistical heterogeneity, a fixed-effects model was used, otherwise a random-effects model was applied as the expected heterogeneity.

## Results

**Figure 1:**
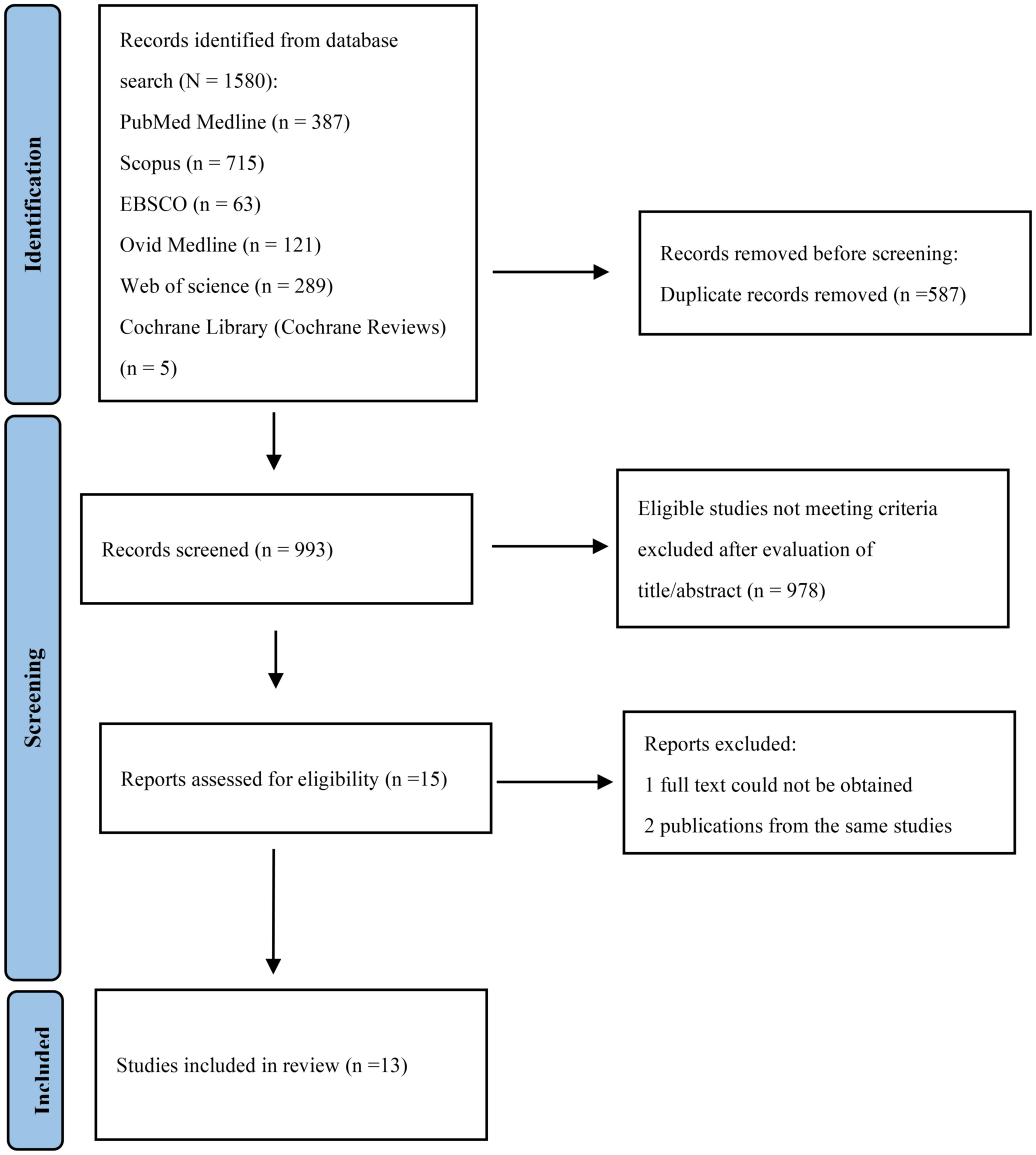
Flow chart depicting the selection of included studies.

The initial searches yielded a total of 1,580 articles: 387 from PubMed Medline, 715 from Scopus, 63 from EBSCO, 121 from Ovid MEDLINE, 289 from Web of Science and 5 from Cochrane Library (Cochrane Reviews). After the removal of 587 duplicates, 993 studies remained. After reading the titles and abstracts, 15 remaining articles were evaluated by full text and one study were excluded because the full article could not be obtained (9). In the case of two different publications of the same studies, the most recent one was included. (10, 11) The flow diagram of the selection process was present in Figure 1.

### Study Characteristics

The characteristics of the 13 studies included in the final analysis were presented in Supplementary Table S2 (11–23). Among them, nine were retrospective case series, and four were prospective studies with only three involving a control group. A total of 128 patients and 445 eyes were identified. For geographical location, seven studies were conducted in Europe, three in America, and three in Asia.

### Quality Assessment

Overall, 2 studies (11, 21) were assigned a level III rating and 12 were assigned a level IV rating. Analysis on risk of bias of included studies were recorded in Supplementary Table S3. The mean MINORS score for non-comparative and comparative studies was 12.00 ± 0.85 and 19.67 ± 0.47, respectively, indicating fair quality of evidence for non-randomized studies and high quality for non-randomized studies. However, only one study (16) (7.14%) reported prospective calculation of the study size and none had an unbiased assessment of the endpoints.

### Visual Outcomes

**Figure 2:**
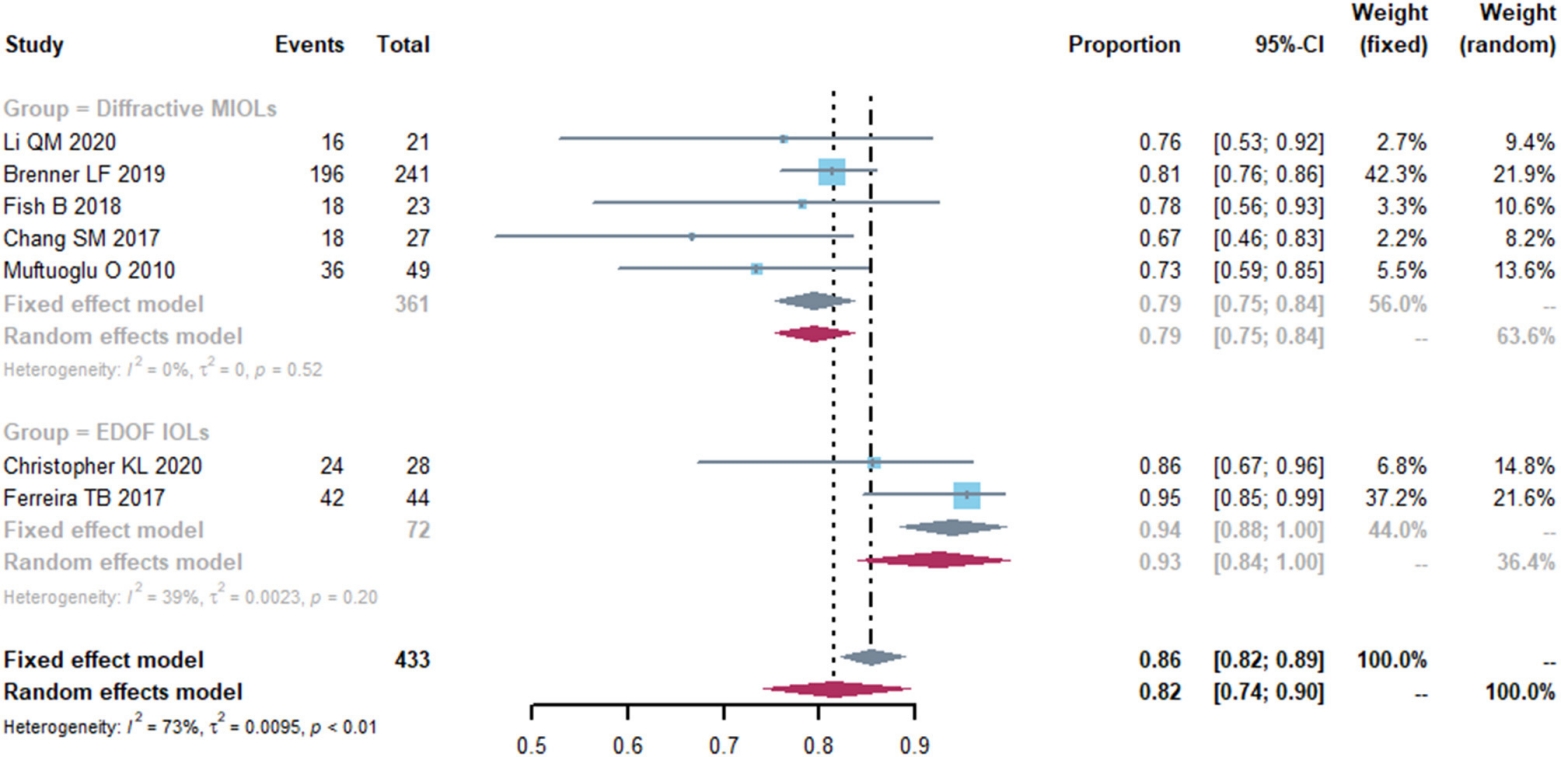
Forest plot of uncorrected distance visual acuity (UDVA). The proportion of the eyes with a postoperative visual acuity ≥ 20/25 was represented in this graph. Events, number of treated eyes with a postoperative visual acuity ≥ 20/25. Total, total number of treated eyes. Proportion, proportion of eyes ≥ 20/25. CI, confidence interval; IOL, intraocular lense; MIOLs, multifocal intraocular lenses; EDOF, extended depth-of-focus.

Seven studies reported uncorrected distance visual acuity (UDVA) and corrected distance visual acuity (CDVA) as outcomes (Supplementary Table S4). The pooled proportion of eyes with postoperative UDVA ≥ 20/25 was 0.82 [95% confidence interval (CI), 0.74-0.90] and the I^2^ was 73% (Figure 2). We performed a subgroup analysis by the IOLs types; presbyopia-correcting IOLs were split into diffractive MIOLs and EDOF IOLs (Figure 2). In diffractive MIOLs group, the I^2^ dropped to 0%, with a proportion of 0.79 (95% CI, 0.75-0.84). In EDOF IOLs group, the pooled proportion was 0.94 (95% CI, 0.88-1.00), which was higher than of the MIOLs group. Next, we conducted further subgroup analyses and divided the diffractive MIOLs into bifocal and trifocal IOLs (Supplementary Figure S1). The proportion in trifocal IOLs group was higher than that in bifocal IOLs group.

**Figure 3:**
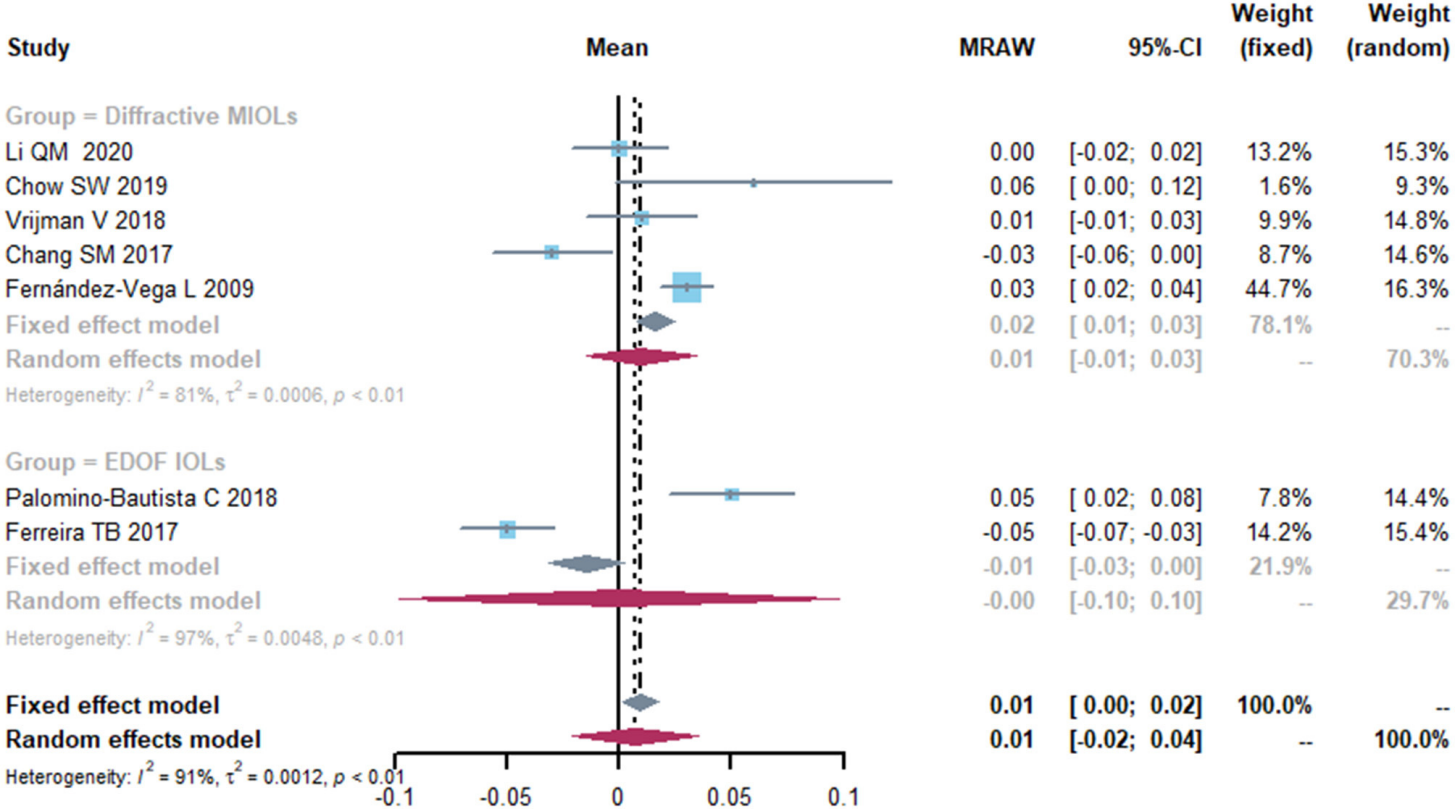
Forest plot of corrected distance visual acuity (CDVA) in logMAR. IOL, intraocular lense; MIOLs, multifocal intraocular lenses; EDOF, extended depth-of-focus; MRAW, the row mean from the study.

The mean CDVA was 0.01 logMAR (95% CI, −0.02-0.04) (Figure 3). The I^2^ was 91%, indicating a large heterogeneity across included studies, thus sensitivity analyses were conducted by omitting one study at a time. Then we performed a subgroup analysis by different types of IOL implanted in cataract surgery. Respectively, 4 and 3 studies reported on diffractive MIOLs and EDOF IOL. The mean CDVA was 0.01 logMAR (95% CI, −0.01-0.03) and −0.00 logMAR (95% CI, −0.10-0.10), respectively. However, although the final results were stable, there was significant heterogeneity which we were unable to eliminate.

Only three studies provided data on uncorrected intermediate visual acuity (UIVA) (Supplementary Table S4). The mean UIVA in the studies by Li et al. (13) and Chow et al. (15) were 0.10 ± 0.10 logMAR and 0.22 ± 0.15 logMAR.

**Figure 4:**
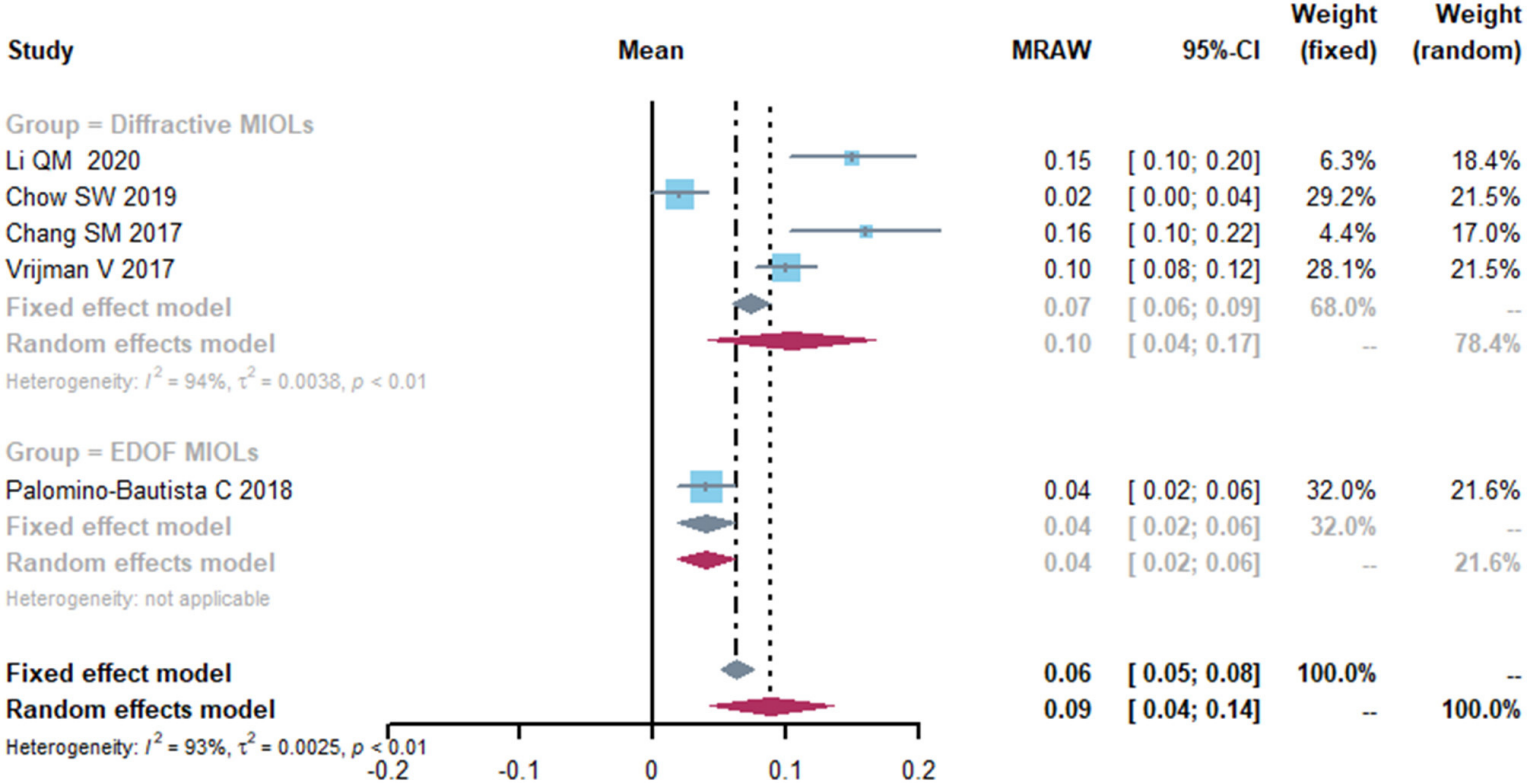
Forest plot of uncorrected near visual acuity (UNVA) in logMAR. IOL, intraocular lense; MIOLs, multifocal intraocular lenses; EDOF, extended depth-of-focus; MRAW, the row mean from the study.

Five studies reported the data on mean postoperative uncorrected near visual acuity (UNVA) (Figure 4). The mean UNVA was 0.09 logMAR (95% CI, 0.04-0.14) and the I^2^ was 93%. Although different subgroup and sensitivity analyses were performed, the source of heterogeneity remained unidentifiable.

### Spectacle Independence

**Figure 5:**
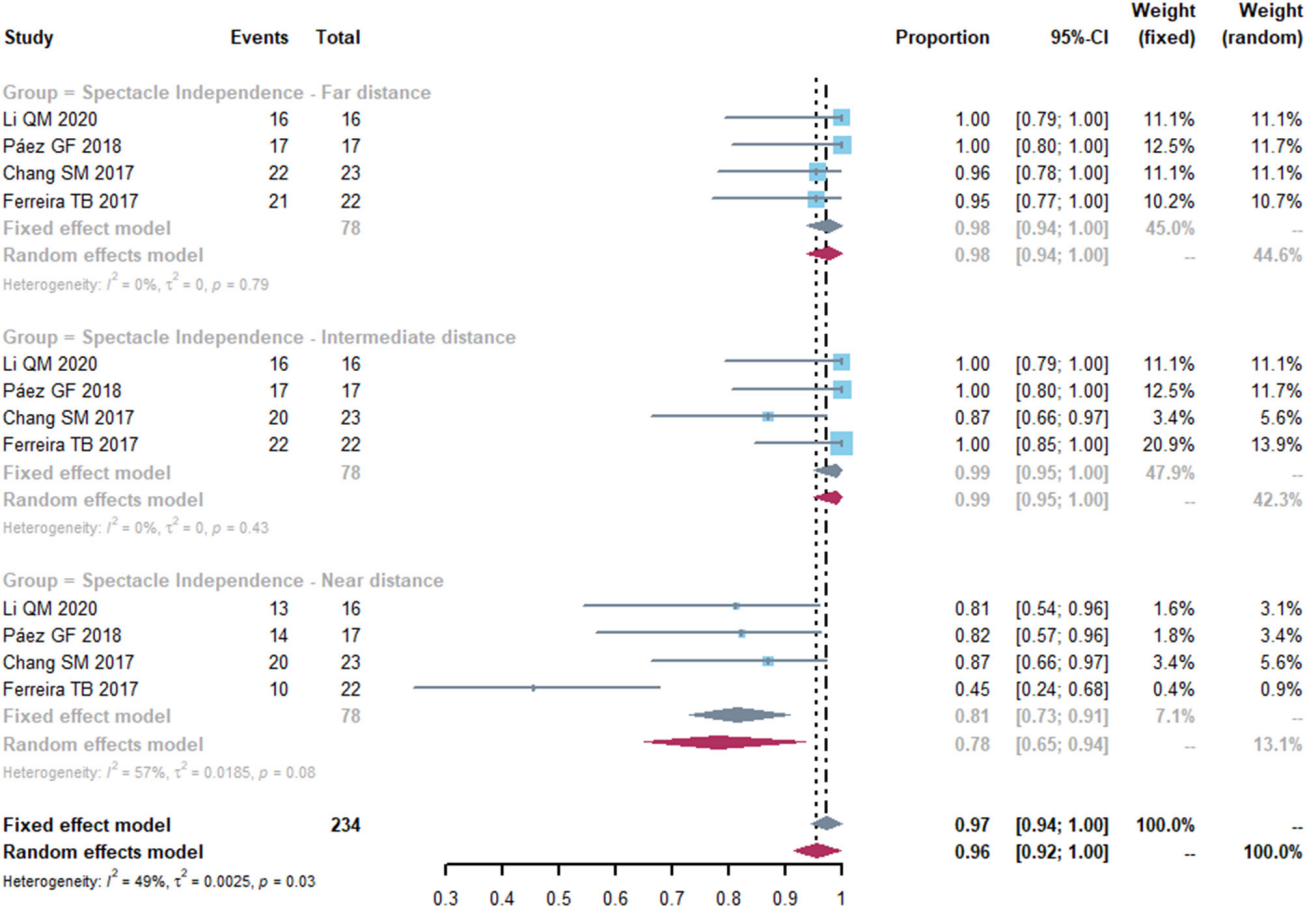
Forest plot of spectacle independence of far, intermediate and near distance. Total, total number of treated eyes. Proportion, proportion of eyes achieved spectacle independence at far, intermediate and near distance.

Four studies reported spectacle independence of far, intermediate and near distance. At far and intermediate distances, no heterogeneity was detected in spectacle independence (Figure 5). The pooled proportion of spectacle independence were 0.98 (95% CI, 0.94-1.00) and 0.99 (95% CI, 0.95-1.00) for far and intermediate distance. However, less patients achieved spectacle independence at near distance, with a proportion of 0.78 (95% CI, 0.65-0.94). We conducted sensitivity analyses and found that after excluding study of Ferreira et al. (21), the heterogeneity decreased (from 57 to 0%), with a proportion of 0.84 (95% CI, 0.75-0.94) (Supplementary Figure S2).

### Visual Quality

**Figure 6:**
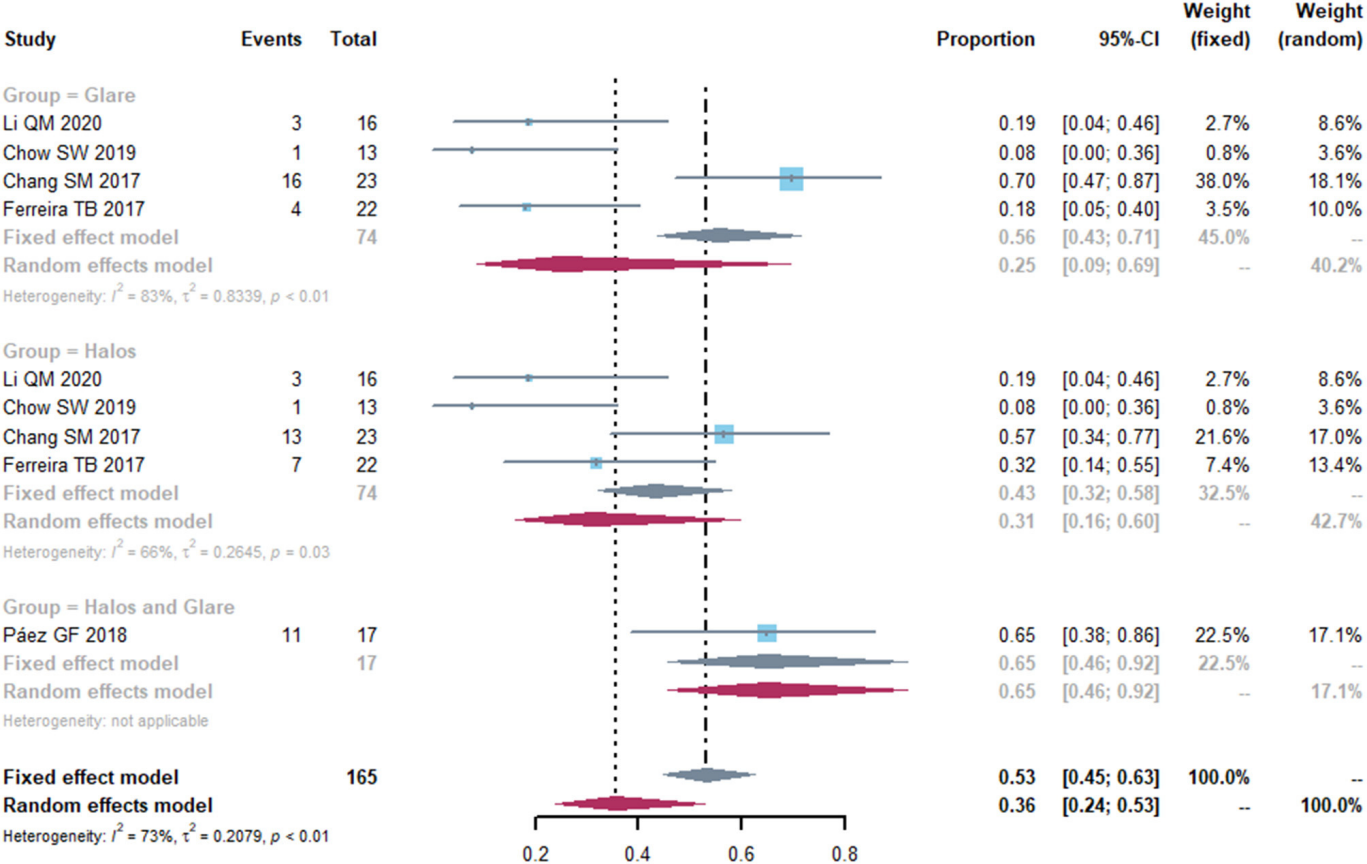
Forest plot of photic disturbance of halos and glare. Total, total number of the patients. Proportion, proportion of patients who suffered from halos and glare.

We identified five studies that evaluated visual quality such as halos and glare (Figure 6). The pooled proportion of patients who suffered from halos was 0.31 (95% CI, 0.16-0.60) and the percentage of participants who were troubled with glare was 0.25 (95% CI, 0.09-0.69). High heterogeneity was found in the halos and glare effects (I^2^ = 66%, *P* = 0.30 and I^2^ = 83%, *P* < 0.01, respectively). Thus, we performed a sensitivity analysis to analyze the sources of heterogeneity. After removing the study by Chang et al. (20), the heterogeneity decreased (I^2^ = 17%, *P* = 0.30 and I^2^ = 0%, *P* = 0.69) in each group Supplementary Figure S3).

### Refractive Outcomes

**Figure 7:**
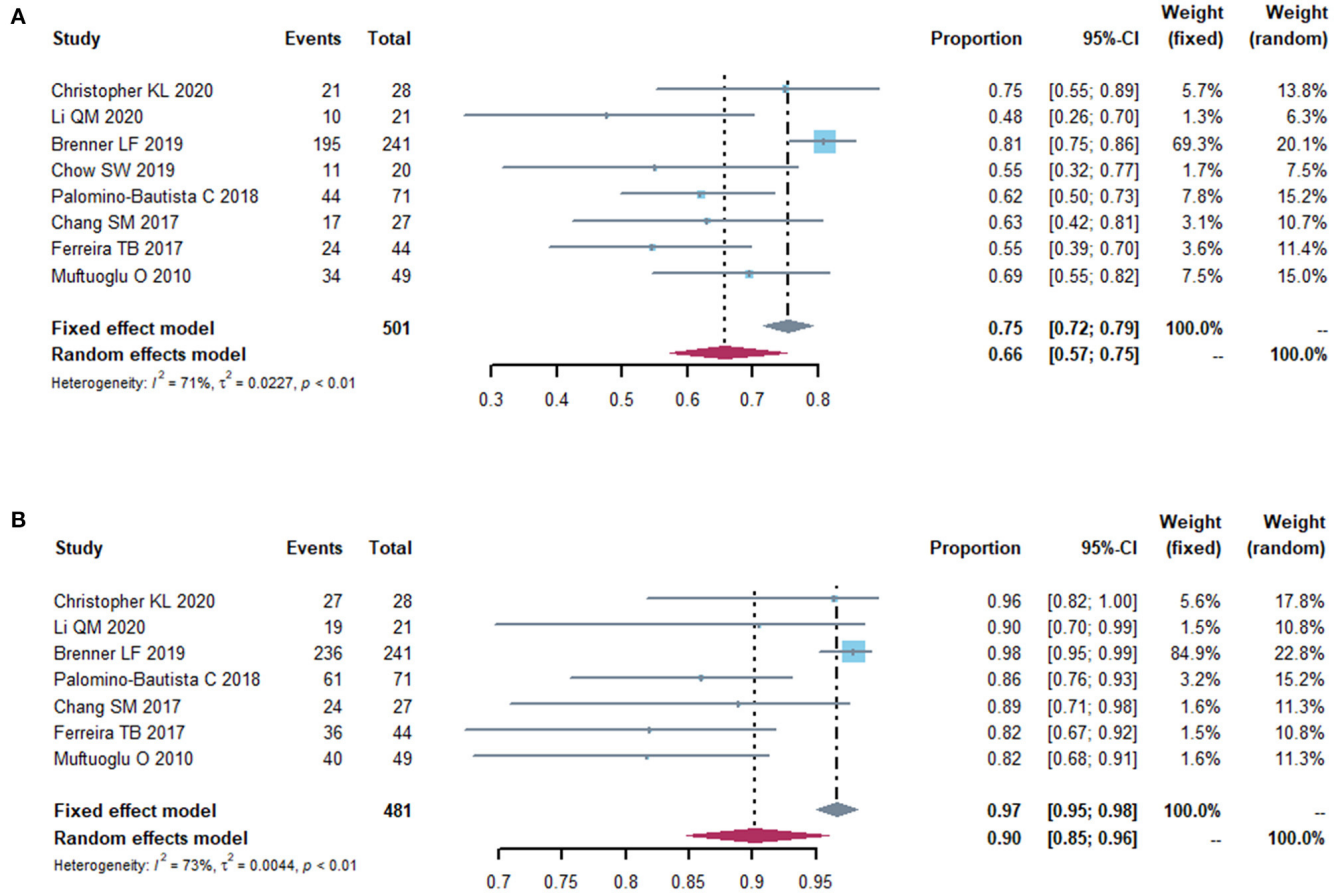
Forest plot of postoperative refraction. The proportion of eyes with a postoperative refraction of 0.5 diopters **(A)** and 1.0 diopters **(B)** from the target refraction.

The postoperative spherical equivalent (SE) was recorded in 11 studies. Eight studies reported postoperative SE within ±0.5D from the targeted refraction and seven within ± 1.0D (Figure 7). The proportion of treated eyes with a postoperative refraction of ±0.5D and ±1.0D within the target refraction was 0.66 (95% CI, 0.57-0.75) and 0.90 (95% CI, 0.85-0.96), respectively. We did a sensitivity analysis, and it turned out that, after removing the study by Brenner et al. (14) the I^2^ dropped to 8 and 40%, with a proportion of 0.64 (95% CI: 0.58, 0.70) and 0.90 (95% CI: 0.86, 0.94) (Supplementary Figure S4). Then, we did a subgroup analysis, splitting the studies into two subgroups according to the follow-up time (<6 months vs. ≥6 months) (Supplementary Figure S4). Two studies that did not provide the follow-up time were excluded in this subgroup analysis. No heterogeneity was detected in 2 subgroups (I^2^ = 0). In the studies with 6-month or more follow-up time, the proportion of postoperative spherical equivalent within ± 0.5D from the targeted refraction was higher (0.66, 95% CI, 0.57-0.76) than that in the studies with follow-up time <6 months (0.58, 95% CI, 0.50-0.67), while the incidence of postoperative spherical equivalent within ±1.0D was similar in both groups.

We also did a subgroup analysis by mean axial length (<26 vs. ≥26 mm) and identified no heterogeneity (I^2^ = 0) between each group (Supplementary Figure S5). The pooled proportion of eyes within ±0.5D from the target refraction in the group with mean axial length <26 mm (0.52, 95% CI, 0.38-0.69) was lower than that in the other group (0.65, 95% CI, 0.58-0.73).

## Discussion

Cataract patients with previous corneal refractive surgery are eager for spectacle independence and a high visual quality after cataract surgery. One of the major defects of monofocal intraocular lenses (IOLs) as replacements for human crystalline lenses is the fixed focus of the IOLs. Compared to monofocal IOLs, presbyopia-correcting IOLs provide wider range of vision and continuation of spectacle independence for distance, intermediate and near vision in post-myopic or post-hyperopic refractive surgery eyes. Many types of presbyopia-correcting IOLs are now available, including multifocal IOLs (MIOLs), extended depth-of-focus (EDOF) IOLs and accommodating IOLs. To date, there has been no systematic review and meta-analysis conducted on this topic.

### Efficacy Analysis

The distance, intermediate and near visual acuity and spectacle independence are the most important endpoints for satisfaction in patients with previous corneal refractive surgery. For distance vision, we performed a meta-analysis on the proportion for UDVA of 20/25 or better in post refractive surgery eyes and found better results in EDOF IOLs group than diffractive MIOLs group. After splitting the diffractive MIOLs into bifocal and trifocal IOLs, the trifocal IOLs showed an improvement in UDVA when compared to bifocal IOLs. However, no significant differences were identified between trifocal and bifocal IOLs for distant VA in patients without previous corneal refractive surgery (24). Thus, further studies are required to demonstrated this clearly. In terms of CDVA, Fernández-Vega et al. (11) and Chow et al. (15) reported an improvement in CDVA after MIOLs implantation in post-myopic LASIK patients. In the present study, although diffractive MIOLs yield better CDVA than EDOF IOL, the result would not be credible to conclude that MIOLs provided better distance vision than EDOF IOL in such cases due to the high heterogeneity and limited number of available studies.

With regards to intermediate and near vision, Ferreira et al. (21) found that the uncorrected near and intermediate visual acuities were better after implantation of EDOF IOL than monofocal IOL, which was in accordance with those results in eyes without previous corneal refractive surgery (25, 26). They reported binocular UIVA and UNVA of 0.1 logMAR or better in 100% and 59.09% of eyes in EDOF IOLs group, and 59.09% and 0% in monofocal group, indicating that EDOF IOLs implanted in eyes that had previous LASIK was able to restore the intermediate and near visual function. In a study by Chang et al. (20), binocular post-operative UIVA and UNVA values of 0.1 logMAR or better were 60.87 and 34.78% of eyes, respectively, in eyes implanted with the diffractive bifocal Tecnis ZMA00 and ZMB00 MIOLs. In eyes without previous corneal refractive surgery, multifocal IOLs presented better near visual acuity while EDOF IOLs showed better results in intermediate vision. However, the present study's results showed better mean UNVA in eyes with EDOF IOLs than diffractive multifocal IOLs. Considering the limited number of studies available and the high between-study heterogeneity, our evidence of intermediate and near VA was insufficient to reach a definitive conclusion. Further research comparing MIOLs and EDOF IOLs in patients with previous corneal refractive surgery is warranted.

In present study, MIOLs and EDOF IOLs performed a good result of spectacle independence for far and intermediate distances. At near distance, the proportion of spectacle dependence increased (from 0.78 to 0.84) and heterogeneity decreased after omitting the study by Ferreira et al. (21), who investigated the clinical outcomes of EDOF IOLs in eyes with previous myopic LASIK. Therefore, it is indicated the postoperative level in dependence for near vision was possibly higher in the EDOF IOLs than the diffractive MIOLs. It is also consistent with the fact that EDOF IOLs, with an extended far focus area which reaches to the intermediate distance, restore distance and intermediate visual function but with restraint of near vision (27–29). In Ferreira et al.'s study (21), more patients implanted with EDOF IOLs were free from glasses than those receiving monofocal IOLs. Although there were no studies comparing MIOLs and monofocal IOLs in eyes with previous corneal surgery, it is proved that MIOLs performed far better than monofocal IOLs on spectacle independence in eyes without previous corneal surgery (30).

### Safety Analysis

It has been reported that the frequency of phenomena including halo, glare, and difficulty in night vision in patients who underwent successful corneal refractive surgery is increased, which are related to excessive postoperative higher order aberration values (19). Studies show that myopic and hyperopic ablation significantly increased corneal higher-order aberration (HOAs), inducing positive and negative spherical aberration (SA) respectively (31). Presbyopia-correcting IOLs with enhanced asphericity may be more appropriate for post-myopic surgery eyes (11, 32, 33). The spherical aberration–correcting Tecnis MIOLs (Johnson & Johnson Vision, Inc.) has −0.27 μm SA and aims to correct the total amount of corneal SA (34). ReSTOR (Alcon Laboratories Inc.) SN6AD1 MIOLs corrects for 0.1 μm of spherical aberration while the SN60D3 does not correct for any (35). The AT LISA MIOLs (Carl Zeiss Meditec AG) and FineVision IOL (PhysIOL) has −0.18 and −0.11 μm SA, respectively. Conversely, eyes that underwent hyperopic LASIK should be implanted with a spherical IOL (36).

In addition to the introduction of HOAs after laser refractive surgery, the position and functional deviation of intraocular lenses might also contribute to photic phenomena such as halos and glare. The capsule in high myopic patients who underwent refractive laser surgery was larger compared to normal patients and the stability of IOLs is slightly worse, which are more likely to lead to photopic side effects (13).

To understand the visual quality obtained with different types of IOLs, we analyzed the incidence of halos and glare in five studies. Ferreira et al. (21) found no significantly difference in the incidence of halos and glare between EDOF IOLs and monofocal IOLs implanted in eyes with previous LASIK. Further decrease in heterogeneity was obtained in the sensitivity analyses, which suggested that the heterogeneity might come from the study by Chang et al. (20). One possible explanation is that the study reported patients who had at least a very mild severity of visual symptoms, increasing the proportion of patients with halos and glare. Spherical aberration is a consideration when choosing the presbyopia-correcting IOLs in eyes that have undergone myopic and hyperopic refractive surgery (37). To date, no explicit guidance exists to preclude the use of presbyopia-correcting IOLs regarding the amount of spherical aberrations or other HOAs. It is suggested that potential MIOLs contraindication thresholds for total HOAs was >2.0 SD (38).

Previous studies showed patients with corneal refractive surgery are more prone to have degradation in contrast sensitivity (9). Among 13 included studies, only two studies provided the result of CS. Chang et al. (20) found no significant difference in CS of the operated eyes with Tecnis MIOLs from that of the unoperated eyes]. However, CS at spatial frequency of 3 cpd in the operated eyes was worse in comparison of their previous study of the Tecnis MIOLs in eyes with virgin cornea, suggesting that visual quality after MIOLs implantation could be affected by LASIK attributed to the increased SA (20). In the study by Ferreira et al. (21), no significant difference between EDOF IOLs and monofocal IOLs implanted in eyes with previous LASIK were found for any spatial frequency evaluated, similarly to the results of Pedrotti et al. (26), in eyes without previous LASIK. The authors concluded that there are no significant differences in terms of distance visual degradation between the monofocal IOLs and EDOF IOLs, confirming the good optical performance of this EDOF IOLs implanted in post- LASIK eyes.

Alternative optical quality parameters are used to quantify visual quality after presbyopia-correcting IOLs implantation in eyes that have had refractive surgery, including the modulation transfer function (MTF) cutoff frequency, the objective scatter index (OSI) and the strehl ratio (SR). MTF is obtained by Fourier transform from the point spread function (PSF) and MTF cutoff is used to express visual quality, referring to the frequency at which the MTF reaches 1% contrast (39). OSI is defined as the amount of the light outside the double-pass retinal intensity PSF image in the peripheral zone vs. that on the central zone (40). SR is calculated as the ratio of the peak focal intensities in aberrated PSF and ideal PSF, reflecting the retinal imaging quality with a value between 0 and 1 (41). The higher MTF cut-off values and the lower OSI values indicate better optical quality. The closer SR is to 1, the smaller the aberration of the eye. Camps et al. stimulated optical performance of three presbyopia-correcting IOLs through MTF and reported the worsening of the ocular optical quality at near and intermediate distances as the pupil increased (42). They demonstrated that the Mini Well and Symfony IOLs worked better than the Mplus IOL in eyes with previous myopic LASIK, and the Mini Well IOL provided acceptable results in eyes with previous myopic LASIK. However, none of the included studies in our paper presented the results of MTF, OSI or SR. Thus, further clinical studies performing the measurement of these parameters are needed in the future.

### Predictability Analysis

Presbyopia-correcting IOLs implantation in cases with previous corneal refractive surgery could be challenging for the IOL power calculations attributed primarily to two factors: (1) inaccurate measurement of the true total corneal refractive power given by keratometers and corneal topography systems (43), (2) incorrect calculation of the effective lens position (ELP) by third- or fourth-generation formulas through an estimation based on the postoperative corneal powers (44, 45). Over the past two decades, investigators have made great effort to present different formulas and keratometry measurements to overcome this problem and provide more predictable outcomes (46). There are mainly two categories of methods for IOL power calculations after corneal refractive surgery. Formulas that required perioperative data and information about the refractive change, which have been proved inaccurate and not helpful in improving the predictability of outcomes (47). The other is the formula independent on the historical information. Currently, there are three major methods in IOL power calculations for eyes without clinical data, including the American Society of Cataract and Refractive Surgery (ASCRS) online calculator, the Barrett True-K formula and the OCT-based IOL calculation formula. Wang et al. suggested that the OCT and True-K No History (TKNH) were promising formulas for post-corneal refractive surgery eyes, which were included in the latest update of the ASCRS IOL calculator (48). For cataract eyes with previous LASIK or PRK, Barrett True-K formula provided more stable predictions than alternative methods (18, 49). Similar results were obtained in a prospective study on the IOL calculations in patients undergoing cataract surgery after SMILE (50). In eyes with previous RK lacking historical data, TKNH and Haigis formulae were recommended for IOL calculation (51).

In this meta-analysis, the postoperative percentages of eyes with expected spherical equivalent within ±0.5 D and ±1.0 D of plano in the study by Brenner et al. (14) was higher and comparable to results in untreated cornea (5). They concluded that the possible reasons for the higher accuracy in their study than others were the exclusion of corneas with abnormal optics and the ablation profiles with better transitions zones, which made the *K*-values for IOL power calculations more reliable. It is demonstrated that the myopia status before laser refractive surgery had an effect on the treatment outcomes (52). In treatments with higher amounts of myopia correction, efficacy measures including accuracy and predictability tended to be lower, which in turn impacted postoperative outcomes of IOL implantation (53, 54).

Total keratometry (TK) from IOLMaster 700 (Carl Zeiss Meditec AG, Jena, Germany) offer an advantage in measuring anterior and posterior corneal curvatures together with corneal thickness in patients whose anterior to posterior corneal relationships are altered, such as post laser surgery (55). It has been proved that the accuracy of formulas combined with TK was comparable to the existing no-history post-myopic laser refractive surgery formulas (56). Although there has been progress in IOL power calculation for presbyopia-correcting IOLs over the past few years, the difficulties still remain. Considering the tolerance to residual refractive error among different types of IOLs, EDOF IOLs may be preferable in post-laser surgery eyes.

In eyes with previous corneal refractive laser surgery for high axial myopia, the IOL calculation have become even more difficult. AL measurement is one of the most influential parameters contributing to the deviation between actual diopter and prediction of diopter after cataract surgery (57, 58). In this study, the predictability percentage of eyes with residual refractive error within 0.5D from the target refraction had a lower value in studies with longer axial length, which was in consistent with the study by Zhou et al. evaluating the accuracy of the refractive prediction in highly myopic eyes without previous refractive surgery (59). Therefore, in patients who have underwent previous corneal refractive laser surgery for high myopia, the axial length of the eyes should be measured by appropriate prediction formula to reduce the refractive error absolute value. A recent study demonstrated that both Barrett True-K No History and SToP (SRK/T) were performed well in calculating EDOF IOLs power in eyes with a history of myopic LASIK/PRK surgery when AL ≥ 28.0 mm (60).

According to previous studies and clinical experience, the refractive status could be stabilized at 3 months postoperatively (61). Therefore, we pooled the data reported at the end of follow-up for subgroup analysis. After splitting the studies into two subgroups, the results suggested that the differences among the various studies may be related to follow-up time. In general, the refractive outcomes should be interpreted in the context of different follow-up durations and it is possible that refractive predictability may become better with longer follow-up.

The limitations of this meta-analysis stem from the quality of evidence available due to the scarcity of prospective or randomized controlled studies on this topic. The majority of included studies were Level IV evidence (84.62%) and only 2 (15.38%) studies were Level III. Additionally, the number of included studies was relatively small, and few studies measured and reported the same outcomes consistently, leading to the difficulties for the credibility of some results. Finally, we attempted to diminish the impact of heterogeneity (e.g., types of IOLs, follow-up time, etc.) by performing sensitivity analysis, but the effect of heterogeneity still could not be eliminated completely.

## Conclusions

Overall, the presbyopia-correcting IOLs were effective at providing satisfactory visual outcomes at near, intermediate and far distance and wider range of spectacle independence in eyes with previous corneal refractive surgery. Although presbyopia-correcting IOLs in eyes with previous corneal laser correction was safe, they inevitably generated more photic effects in the form of halos and glare and decreased in contrast sensitivity. Considering the induction of spherical aberration by corneal laser surgery, eyes that underwent myopic ablation should be implanted with aspheric IOLs and spherical IOLs for hyperopic ablation. The progress in IOL power calculations for presbyopia-correcting IOLs and formulas combined with total keratometry (TK) provide accurate predictability for achieving a better refractive result within ±0.5D and ±1.0D of target refraction. More evidence-based publications and RCTs, making a comparison between presbyopia-correcting IOLs and monofocal IOLs or among different types of the presbyopia-correcting IOLs, are warranted to provide guidelines for IOLs selection in patients who have had corneal refractive surgery in the future.

## Data Availability Statement

The original contributions presented in the study are included in the article/Supplementary Material, further inquiries can be directed to the corresponding author/s.

## Author Contributions

YJ was responsible for conception and design. YS and YH collected the literatures, extracted and analyzed the data. YS prepared the final manuscript. XR was involved in reviewing of the manuscript. All authors contributed to the article and approved the submitted version.

## Funding

This work was supported by the National Natural Science Foundation of China (Grant Nos. 81770907 and 82070942) and Shanghai Talent Development Fund (Grant No. 2018049).

## Conflict of Interest

The authors declare that the research was conducted in the absence of any commercial or financial relationships that could be construed as a potential conflict of interest.

## Publisher's Note

All claims expressed in this article are solely those of the authors and do not necessarily represent those of their affiliated organizations, or those of the publisher, the editors and the reviewers. Any product that may be evaluated in this article, or claim that may be made by its manufacturer, is not guaranteed or endorsed by the publisher.
