# Supplementary material for: Presbyopia-Correcting Intraocular Lenses Implantation in Eyes After Corneal Refractive Laser Surgery: A Meta-Analysis and Systematic Review
Source: Front Med (Lausanne). 2022 Apr 11;9:834805. doi: 10.3389/fmed.2022.834805 (PMC9035540; doi:10.3389/fmed.2022.834805)
Supplement: Supplementary file 1 [file Table_1.DOCX]

| **Supplemental Table S1.** Search strategy for electronic databases. | | |
| --- | --- | --- |
| **Database** | **Search strategy** | **Results** |
| PubMed | (((((((((((((Capsule Opacification) OR (Capsule Opacifications)) OR (Opacification, Capsule)) OR (Opacifications, Capsule)) OR (Secondary Cataract)) OR (Cataract, Secondary)) OR (Cataracts, Secondary)) OR (Secondary Cataracts)) OR (((((((((((cataract) OR (Lens Opacities)) OR (Lens Opacity)) OR (Opacities, Lens)) OR (Opacity, Lens)) OR (Cataract, Membranous)) OR (Cataracts, Membranous)) OR (Membranous Cataract)) OR (Membranous Cataracts)) OR (Pseudoaphakia)) OR (Pseudoaphakias))) OR ((((((((Capsule Opacification) OR (Capsule Opacifications)) OR (Opacification, Capsule)) OR (Opacifications, Capsule)) OR (Secondary Cataract)) OR (Cataract, Secondary)) OR (Cataracts, Secondary)) OR (Secondary Cataracts))) OR (((((((((((cataract) OR (Lens Opacities)) OR (Lens Opacity)) OR (Opacities, Lens)) OR (Opacity, Lens)) OR (Cataract, Membranous)) OR (Cataracts, Membranous)) OR (Membranous Cataract)) OR (Membranous Cataracts)) OR (Pseudoaphakia)) OR (Pseudoaphakias))) AND (((((((((Phakic Intraocular Lenses) OR (Intraocular Lenses, Phakic)) OR (Lenses, Phakic Intraocular)) OR (Lens, Intraocular, Phakic)) OR (Phakic Intraocular Lens)) OR (Intraocular Lens, Phakic)) OR (Lens, Phakic Intraocular)) OR (((((((((((((Multifocal Intraocular Lens) OR (Intraocular Lens, Multifocal)) OR (Multifocal IOLs)) OR (IOL, Multifocal)) OR (Multifocal IOL)) OR (Accommodating Intraocular Lenses)) OR (Intraocular Lenses, Accommodating)) OR (Accommodating Intraocular Lens)) OR (Intraocular Lens, Accommodating)) OR (Accommodating IOLs)) OR (Accommodating IOL)) OR (IOL, Accommodating)) OR (IOLs, Accommodating))) OR (((((((Lenses, Intraocular) OR (Intraocular Lenses)) OR (Lens, Intraocular)) OR (Intraocular Lens)) OR (Implantable Contact Lens)) OR (Contact Lens, Implantable)) OR (Lens, Implantable Contact)))) AND (((((((((((((Laser Corneal Surgeries) OR (Surgeries, Laser Corneal)) OR (Surgery, Laser Corneal)) OR (Laser Corneal Surgery)) OR (Keratectomy, Laser)) OR (Keratectomies, Laser)) OR (Laser Keratectomies)) OR (Laser Keratectomy)) OR (Photokeratectomy)) OR (Photokeratectomies)) OR (((((((((((((((((((((Laser Subepithelial Keratomileusis) OR (Subepithelial Keratomileusis, Laser)) OR (LASEK)) OR (Laser-Assisted Sub-Epithelial Keratomileusis)) OR (Sub-Epithelial Keratomileusis, Laser-Assisted)) OR (Keratomileusis, Subepithelial, Laser-Assisted)) OR (Subepithelial Keratomileusis, Laser-Assisted)) OR (Laser-Assisted Subepithelial Keratomileusis)) OR (Subepithelial Keratomileusis, Laser Assisted)) OR (Subepithelial Photorefractive Keratectomy)) OR (Keratectomies, Subepithelial Photorefractive)) OR (Keratectomy, Subepithelial Photorefractive)) OR (Photorefractive Keratectomies, Subepithelial)) OR (Photorefractive Keratectomy, Subepithelial)) OR (Subepithelial Photorefractive Keratectomies)) OR (Laser-Assisted Subepithelial Keratectomy)) OR (Laser Assisted Subepithelial Keratectomy)) OR (Laser-Assisted Subepithelial Keratectomies)) OR (Subepithelial Keratectomies, Laser-Assisted)) OR (Subepithelial Keratectomy, Laser-Assisted)) OR (Subepithelial Keratectomy, Laser Assisted))) OR ((((((((Laser-Assisted Stromal In Situ Keratomileusis) OR (Laser Assisted Stromal In Situ Keratomileusis)) OR (Laser Intrastromal Keratomileusis)) OR (Intrastromal Keratomileuses, Laser)) OR (Intrastromal Keratomileusis, Laser)) OR (Laser Intrastromal Keratomileuses)) OR (Laser In Situ Keratomileusis)) OR (LASIK))) OR (((Keratectomies, Photorefractive) OR (Keratectomy, Photorefractive)) OR (Photorefractive Keratectomies)))) AND ("Study Characteristics" [Publication Type]) | 387 |
| Scopus | (TITLE-ABS-KEY (laser AND corneal AND surgeries)  OR  TITLE-ABS-KEY (surgeries AND laser AND corneal)  OR  TITLE-ABS-KEY (surgery AND laser AND corneal)  OR  TITLE-ABS-KEY (laser AND corneal AND surgery)  OR  TITLE-ABS-KEY (keratectomy AND laser)  OR  TITLE-ABS-KEY (keratectomies AND laser)  OR  TITLE-ABS-KEY (laser AND keratectomies)  OR  TITLE-ABS-KEY (laser AND keratectomy)  OR  TITLE-ABS-KEY (photokeratectomy)  OR  TITLE-ABS-KEY (photokeratectomies)  OR  TITLE-ABS-KEY (laser AND subepithelial AND keratomileusis)  OR  TITLE-ABS-KEY (subepithelial AND keratomileusis AND laser)  OR  TITLE-ABS-KEY (lasek)  OR  TITLE-ABS-KEY (laser-assisted AND sub-epithelial AND keratomileusis)  OR  TITLE-ABS-KEY (sub-epithelial AND keratomileusis AND laser-assisted)  OR  TITLE-ABS-KEY (keratomileusis AND subepithelial AND laser-assisted)  OR  TITLE-ABS-KEY (subepithelial AND keratomileusis AND laser-assisted)  OR  TITLE-ABS-KEY (laser-assisted AND subepithelial AND keratomileusis)  OR  TITLE-ABS-KEY (subepithelial AND keratomileusis AND laser AND assisted)  OR  TITLE-ABS-KEY (subepithelial AND photorefractive AND keratectomy)  OR  TITLE-ABS-KEY (keratectomies AND subepithelial AND photorefractive)  OR  TITLE-ABS-KEY (keratectomy AND subepithelial AND photorefractive)  OR  TITLE-ABS-KEY (photorefractive AND keratectomies AND subepithelial)  OR  TITLE-ABS-KEY (photorefractive AND keratectomy AND subepithelial)  OR  TITLE-ABS-KEY (subepithelial AND photorefractive AND keratectomies)  OR  TITLE-ABS-KEY (laser-assisted AND subepithelial AND keratectomy)  OR  TITLE-ABS-KEY (laser AND assisted AND subepithelial AND keratectomy)  OR  TITLE-ABS-KEY (laser-assisted AND subepithelial AND keratectomies)  OR  TITLE-ABS-KEY (subepithelial AND keratectomies AND laser-assisted)  OR  TITLE-ABS-KEY (subepithelial AND keratectomy AND laser-assisted)  OR  TITLE-ABS-KEY (subepithelial AND keratectomy AND laser AND assisted)  OR  TITLE-ABS-KEY (laser-assisted AND stromal AND in AND situ AND keratomileusis)  OR  TITLE-ABS-KEY (laser AND assisted AND stromal AND in AND situ AND keratomileusis)  OR  TITLE-ABS-KEY (laser AND intrastromal AND keratomileusis)  OR  TITLE-ABS-KEY (intrastromal AND keratomileuses AND laser)  OR  TITLE-ABS-KEY (intrastromal AND keratomileusis AND laser)  OR  TITLE-ABS-KEY (laser AND intrastromal AND keratomileuses)  OR  TITLE-ABS-KEY (laser AND in AND situ AND keratomileusis)  OR  TITLE-ABS-KEY (lasik)  OR  TITLE-ABS-KEY (keratectomies AND photorefractive)  OR  TITLE-ABS-KEY (keratectomy AND photorefractive)  OR  TITLE-ABS-KEY (photorefractive AND keratectomies)) AND  (TITLE-ABS-KEY (capsule AND opacification)  OR  TITLE-ABS-KEY (capsule AND opacifications)  OR  TITLE-ABS-KEY (opacification AND capsule)  OR  TITLE-ABS-KEY (opacifications AND capsule)  OR  TITLE-ABS-KEY (secondary AND cataract)  OR  TITLE-ABS-KEY (cataract AND secondary)  OR  TITLE-ABS-KEY (cataracts AND secondary)  OR  TITLE-ABS-KEY (secondary AND cataracts)  OR  TITLE-ABS-KEY (cataract)  OR  TITLE-ABS-KEY (lens AND opacities)  OR  TITLE-ABS-KEY (lens AND opacity)  OR  TITLE-ABS-KEY (opacities AND lens)  OR  TITLE-ABS-KEY (opacity AND lens)  OR  TITLE-ABS-KEY (cataract AND membranous)  OR  TITLE-ABS-KEY (cataracts AND membranous)  OR  TITLE-ABS-KEY (membranous AND cataract)  OR  TITLE-ABS-KEY (membranous AND cataracts)  OR  TITLE-ABS-KEY (pseudoaphakia)  OR  TITLE-ABS-KEY (pseudoaphakias)  OR  TITLE-ABS-KEY (capsule AND opacification)  OR  TITLE-ABS-KEY (capsule AND opacifications)  OR  TITLE-ABS-KEY (opacification AND capsule)  OR  TITLE-ABS-KEY (opacifications AND capsule)  OR  TITLE-ABS-KEY (secondary AND cataract)  OR  TITLE-ABS-KEY (cataract AND secondary)  OR  TITLE-ABS-KEY (cataracts AND secondary)  OR  TITLE-ABS-KEY (secondary AND cataracts)  OR  TITLE-ABS-KEY (cataract)  OR  TITLE-ABS-KEY (lens AND opacities)  OR  TITLE-ABS-KEY (lens AND opacity)  OR  TITLE-ABS-KEY (opacities AND lens)  OR  TITLE-ABS-KEY (opacity AND lens)  OR  TITLE-ABS-KEY (cataract AND membranous)  OR  TITLE-ABS-KEY (cataracts AND membranous)  OR  TITLE-ABS-KEY (membranous AND cataract)  OR  TITLE-ABS-KEY (membranous AND cataracts)  OR  TITLE-ABS-KEY (pseudoaphakia)  OR  TITLE-ABS-KEY (pseudoaphakias)) AND  (TITLE-ABS-KEY (phakic AND intraocular AND lenses)  OR  TITLE-ABS-KEY (intraocular AND lenses AND phakic)  OR  TITLE-ABS-KEY (lenses AND phakic AND intraocular)  OR  TITLE-ABS-KEY (lens AND intraocular AND phakic)  OR  TITLE-ABS-KEY (phakic AND intraocular AND lens)  OR  TITLE-ABS-KEY (intraocular AND lens AND phakic)  OR  TITLE-ABS-KEY (lens AND phakic AND intraocular)  OR  TITLE-ABS-KEY (multifocal AND intraocular AND lens)  OR  TITLE-ABS-KEY (intraocular AND lens AND multifocal)  OR  TITLE-ABS-KEY (multifocal AND iols)  OR  TITLE-ABS-KEY (iol AND multifocal)  OR  TITLE-ABS-KEY (multifocal AND iol)  OR  TITLE-ABS-KEY (accommodating AND intraocular AND lenses)  OR  TITLE-ABS-KEY (intraocular AND lenses AND accommodating)  OR  TITLE-ABS-KEY (accommodating AND intraocular AND lens)  OR  TITLE-ABS-KEY (intraocular AND lens AND accommodating)  OR  TITLE-ABS-KEY (accommodating AND iols)  OR  TITLE-ABS-KEY (accommodating AND iol)  OR  TITLE-ABS-KEY (iol AND accommodating)  OR  TITLE-ABS-KEY (iols AND accommodating)  OR  TITLE-ABS-KEY (lenses AND intraocular)  OR  TITLE-ABS-KEY (intraocular AND lenses)  OR  TITLE-ABS-KEY (lens AND intraocular)  OR  TITLE-ABS-KEY (intraocular AND lens)  OR  TITLE-ABS-KEY (implantable AND contact AND lens)  OR  TITLE-ABS-KEY (contact AND lens AND implantable)  OR  TITLE-ABS-KEY (lens AND implantable AND contact)) AND  (TITLE-ABS-KEY (clinical AND study)  OR  TITLE-ABS-KEY (clinical AND conference)  OR  TITLE-ABS-KEY (comparative AND study)  OR  TITLE-ABS-KEY (consensus AND development AND conference)  OR  TITLE-ABS-KEY (evaluation AND study)  OR  TITLE-ABS-KEY (meta-analysis)  OR  TITLE-ABS-KEY (multicenter AND study)  OR  TITLE-ABS-KEY (scientific AND integrity AND review)  OR  TITLE-ABS-KEY (systematic AND review)  OR  TITLE-ABS-KEY (twin AND study)  OR  TITLE-ABS-KEY (validation AND study)  OR  TITLE-ABS-KEY (case AND reports)  OR  TITLE-ABS-KEY (clinical AND trial)  OR  TITLE-ABS-KEY (clinical AND trial AND protocol)  OR  TITLE-ABS-KEY (clinical AND trial AND veterinary)  OR  TITLE-ABS-KEY (observational AND study)  OR  TITLE-ABS-KEY (observational AND study AND veterinary)  OR  TITLE-ABS-KEY (randomized AND controlled AND trial AND veterinary)  OR  TITLE-ABS-KEY (adaptive AND clinical AND trial)  OR  TITLE-ABS-KEY (clinical AND trial AND phase AND i)  OR  TITLE-ABS-KEY (clinical AND trial AND phase AND ii)  OR  TITLE-ABS-KEY (clinical AND trial AND phase AND iii)  OR  TITLE-ABS-KEY (clinical AND trial AND phase AND iv)  OR  TITLE-ABS-KEY (controlled AND clinical AND trial)  OR  TITLE-ABS-KEY (randomized AND controlled AND trial)) | 715 |
| Ovid Medline | 1 (cataract or cataract, membranous or cataracts or cataracts, membranous or lens opacities or lens opacity or membranous cataract or membranous cataracts or opacities, lens or opacity, lens or pseudoaphakia or pseudoaphakias).mp.  2 (corneal surgeries, laser or corneal surgery, laser or keratectomies, laser or keratectomy, laser or laser corneal surgeries or laser corneal surgery or laser keratectomies or laser keratectomy or photokeratectomies or photokeratectomy or surgeries, laser corneal or surgery, laser corneal or (keratectomies, subepithelial photorefractive or keratectomy, subepithelial, laser-assisted or keratectomy, subepithelial photorefractive or keratomileusis, subepithelial, laser-assisted or lasek or laser assisted subepithelial keratectomy or laser subepithelial keratomileusis or laser-assisted sub-epithelial keratomileusis or laser-assisted subepithelial keratectomies or laser-assisted subepithelial keratectomy or laser-assisted subepithelial keratomileusis or photorefractive keratectomies, subepithelial or photorefractive keratectomy, subepithelial or sub-epithelial keratomileusis, laser-assisted or subepithelial keratectomies, laser-assisted or subepithelial keratectomy, laser assisted or subepithelial keratectomy, laser-assisted or subepithelial keratomileusis, laser or subepithelial keratomileusis, laser assisted or subepithelial keratomileusis, laser-assisted or subepithelial photorefractive keratectomies or subepithelial photorefractive keratectomy) or (intrastromal keratomileuses, laser or intrastromal keratomileusis, laser or keratomileusis, laser in situ or lasik or laser assisted stromal in situ keratomileusis or laser in situ keratomileusis or laser intrastromal keratomileuses or laser intrastromal keratomileusis or laser-assisted stromal in situ keratomileusis) or (keratectomies, photorefractive or keratectomy, photorefractive or photorefractive keratectomies or photorefractive keratectomy)).mp.  3 (contact lens, implantable or implantable contact lens or intraocular lens or intraocular lenses or lens, implantable contact or lens, intraocular or lenses, intraocular or (accommodating iol or accommodating iols or accommodating intraocular lens or accommodating intraocular lenses or iol, accommodating or iol, multifocal or iols, accommodating or intraocular lens, accommodating or intraocular lens, multifocal or intraocular lenses, accommodating or multifocal iol or multifocal iols or multifocal intraocular lens or multifocal intraocular lenses) or (intraocular lens, phakic or intraocular lenses, phakic or lens, intraocular, phakic or lens, phakic intraocular or lenses, phakic intraocular or phakic intraocular lens or phakic intraocular lenses)).mp.  4 Study Characteristics/ or Case Reports/ or Clinical Conference/ or Clinical Study/ or Clinical Trial/ or Clinical Trial Protocol/ or Clinical Trial, Veterinary/ or Observational Study/ or Observational Study, Veterinary/ or Comparative Study/ or Consensus Development Conference/ or Evaluation Study/ or Meta-Analysis/ or Multicenter Study/ or Scientific Integrity Review/ or Systematic Review/ or Twin Study/ or Validation Study/  5 1 and 2 and 3 and 4 | 121 |
| Web of Science | #1 TS=(Laser Corneal Surgeries) OR TS=(Surgeries, Laser Corneal) OR TS=(Surgery, Laser Corneal) OR TS=(Laser Corneal Surgery) OR TS=(Keratectomy, Laser) OR TS=(Keratectomies, Laser) OR TS=(Laser Keratectomies) OR TS=(Laser Keratectomy) OR TS=(Photokeratectomy) OR TS=(Photokeratectomies) OR TS=(Laser Subepithelial Keratomileusis) OR TS=(Subepithelial Keratomileusis, Laser) OR TS=(LASEK) OR TS=(Laser-Assisted Sub-Epithelial Keratomileusis) OR TS=(Sub-Epithelial Keratomileusis, Laser-Assisted) OR TS=(Keratomileusis, Subepithelial, Laser-Assisted) OR TS=(Subepithelial Keratomileusis, Laser-Assisted) OR TS=(Laser-Assisted Subepithelial Keratomileusis) OR TS=(Subepithelial Keratomileusis, Laser Assisted) OR TS=(Subepithelial Photorefractive Keratectomy) OR TS=(Keratectomies, Subepithelial Photorefractive) OR TS=(Keratectomy, Subepithelial Photorefractive) OR TS=(Photorefractive Keratectomies, Subepithelial) OR TS=(Photorefractive Keratectomy, Subepithelial) OR TS=(Subepithelial Photorefractive Keratectomies) OR TS=(Laser-Assisted Subepithelial Keratectomy) OR TS=(Laser Assisted Subepithelial Keratectomy) OR TS=(Laser-Assisted Subepithelial Keratectomies) OR TS=(Subepithelial Keratectomies, Laser-Assisted) OR TS=(Subepithelial Keratectomy, Laser-Assisted) OR TS=(Subepithelial Keratectomy, Laser Assisted) OR TS=(Laser-Assisted Stromal In Situ Keratomileusis) OR TS=(Laser Assisted Stromal In Situ Keratomileusis) OR TS=(Laser Intrastromal Keratomileusis) OR TS=(Intrastromal Keratomileuses, Laser) OR TS=(Intrastromal Keratomileusis, Laser) OR TS=(Laser Intrastromal Keratomileuses) OR TS=(Laser In Situ Keratomileusis) OR TS=(LASIK) OR TS=(Keratectomies, Photorefractive) OR TS=(Keratectomy, Photorefractive) OR TS=(Photorefractive Keratectomies)  #2 TS=(Capsule Opacification) OR TS=(Capsule Opacifications) OR TS=(Opacification, Capsule) OR TS=(Opacifications, Capsule) OR TS=(Secondary Cataract) OR TS=(Cataract, Secondary) OR TS=(Cataracts, Secondary) OR TS=(Secondary Cataracts) OR TS=(cataract) OR TS=(Lens Opacities) OR TS=(Lens Opacity) OR TS=(Opacities, Lens) OR TS=(Opacity, Lens) OR TS=(Cataract, Membranous) OR TS=(Cataracts, Membranous) OR TS=(Membranous Cataract) OR TS=(Membranous Cataracts) OR TS=(Pseudoaphakia) OR TS=(Pseudoaphakias) OR TS=(Capsule Opacification) OR TS=(Capsule Opacifications) OR TS=(Opacification, Capsule) OR TS=(Opacifications, Capsule) OR TS=(Secondary Cataract) OR TS=(Cataract, Secondary) OR TS=(Cataracts, Secondary) OR TS=(Secondary Cataracts) OR TS=(cataract) OR TS=(Lens Opacities) OR TS=(Lens Opacity) OR TS=(Opacities, Lens) OR TS=(Opacity, Lens) OR TS=(Cataract, Membranous) OR TS=(Cataracts, Membranous) OR TS=(Membranous Cataract) OR TS=(Membranous Cataracts) OR TS=(Pseudoaphakia) OR TS=(Pseudoaphakias)  #3 TS=(Phakic Intraocular Lenses) OR TS=(Intraocular Lenses, Phakic) OR TS=(Lenses, Phakic Intraocular) OR TS=(Lens, Intraocular, Phakic) OR TS=(Phakic Intraocular Lens) OR TS=(Intraocular Lens, Phakic) OR TS=(Lens, Phakic Intraocular) OR TS=(Multifocal Intraocular Lens) OR TS=(Intraocular Lens, Multifocal) OR TS=(Multifocal IOLs) OR TS=(IOL, Multifocal) OR TS=(Multifocal IOL) OR TS=(Accommodating Intraocular Lenses) OR TS=(Intraocular Lenses, Accommodating) OR TS=(Accommodating Intraocular Lens) OR TS=(Intraocular Lens, Accommodating) OR TS=(Accommodating IOLs) OR TS=(Accommodating IOL) OR TS=(IOL, Accommodating) OR TS=(IOLs, Accommodating) OR TS=(Lenses, Intraocular) OR TS=(Intraocular Lenses) OR TS=(Lens, Intraocular) OR TS=(Intraocular Lens) OR TS=(Implantable Contact Lens) OR TS=(Contact Lens, Implantable) OR TS=(Lens, Implantable Contact)  #4 TS=(clinical study) OR TS=(Clinical Conference) OR TS=( comparative study) OR TS=( Consensus Development Conference) OR TS=( Evaluation Study) OR TS=( Meta-Analysis) OR TS=( Multicenter Study) OR TS=( Scientific Integrity Review) OR TS=( Systematic Review) OR TS=( Twin Study) OR TS=( Validation Study) OR TS=( Case Reports) OR TS=( Clinical Trial) OR TS=( Clinical Trial Protocol) OR TS=( Clinical Trial, Veterinary) OR TS=( Observational Study) OR TS=( Observational Study, Veterinary) OR TS=( Randomized Controlled Trial, Veterinary) OR TS=( Adaptive Clinical Trial) OR TS=( Clinical Trial, Phase I) OR TS=( Clinical Trial, Phase II) OR TS=( Clinical Trial, Phase III) OR TS=( Clinical Trial, Phase IV) OR TS=( Controlled Clinical Trial) OR TS=( Randomized Controlled Trial)  #5 #4 AND #3 AND #2 AND #1 | 289 |
| Cochrane Library | #1 (Lens Opacity):ti,ab,kw OR (Lens Opacities):ti,ab,kw OR (Opacity, Lens):ti,ab,kw OR (Opacities, Lens):ti,ab,kw OR (Cataract, Membranous):ti,ab,kw (Word variations have been searched) in Cochrane Reviews  #2 (Membranous Cataracts):ti,ab,kw OR (Pseudoaphakias):ti,ab,kw OR (Cataracts, Membranous):ti,ab,kw OR (Pseudoaphakia):ti,ab,kw OR (Membranous Cataract):ti,ab,kw (Word variations have been searched) in Cochrane Reviews  #3 (Cataracts):ti,ab,kw (Word variations have been searched) in Cochrane Reviews  #4 #1 OR #2 OR #3 in Cochrane Reviews  #5 (Surgeries, Refractive):ti,ab,kw OR (Surgical Procedures, Refractive):ti,ab,kw OR (Refractive Surgical Procedure):ti,ab,kw OR (Refractive Surgery):ti,ab,kw OR (Procedure, Refractive Surgical):ti,ab,kw (Word variations have been searched) in Cochrane Reviews  #6 (Surgical Procedure, Keratorefractive):ti,ab,kw OR (Procedures, Keratorefractive Surgical):ti,ab,kw OR (Procedure, Keratorefractive Surgical):ti,ab,kw OR (Keratorefractive Surgical Procedure):ti,ab,kw OR (Surgical Procedures, Keratorefractive):ti,ab,kw (Word variations have been searched) in Cochrane Reviews  #7 (Keratorefractive Surgical Procedures):ti,ab,kw OR (Refractive Surgeries):ti,ab,kw OR (Surgery, Refractive):ti,ab,kw OR (Surgical Procedure, Refractive):ti,ab,kw OR (Procedures, Refractive Surgical):ti,ab,kw (Word variations have been searched) in Cochrane Reviews  #8 #5 OR #6 OR #7 in Cochrane Reviews  #9 (Implantable Contact Lens):ti,ab,kw OR (Lens, Implantable Contact):ti,ab,kw OR (Contact Lens, Implantable):ti,ab,kw OR (Intraocular Lens):ti,ab,kw OR (Intraocular Lenses):ti,ab,kw (Word variations have been searched) in Cochrane Reviews  #10 (Lens, Intraocular):ti,ab,kw OR (Intraocular Lenses, Phakic):ti,ab,kw OR (Phakic Intraocular Lens):ti,ab,kw OR (Lens, Intraocular, Phakic):ti,ab,kw OR (Lenses, Phakic Intraocular):ti,ab,kw (Word variations have been searched) in Cochrane Reviews  #11 (Lens, Phakic Intraocular):ti,ab,kw OR (Intraocular Lens, Phakic):ti,ab,kw OR (Multifocal Intraocular Lens):ti,ab,kw OR (Intraocular Lens, Multifocal):ti,ab,kw OR (Accommodating Intraocular Lenses):ti,ab,kw (Word variations have been searched) in Cochrane Reviews  #12 (Intraocular Lenses, Accommodating):ti,ab,kw OR (Intraocular Lens, Accommodating):ti,ab,kw OR (Accommodating Intraocular Lens):ti,ab,kw OR (Multifocal IOLs):ti,ab,kw OR (IOL, Multifocal):ti,ab,kw (Word variations have been searched) in Cochrane Reviews  #13 (Multifocal IOL):ti,ab,kw OR (IOLs, Accommodating):ti,ab,kw OR (Accommodating IOL):ti,ab,kw OR (IOL, Accommodating):ti,ab,kw OR (Accommodating IOLs):ti,ab,kw (Word variations have been searched) in Cochrane Reviews  #14 #9 OR #10 OR #11 OR #12 OR #13 in Cochrane Reviews  #15 #4 AND #8 AND #14 in Cochrane Reviews | 5 |
| EBSCO | S1 Laser Corneal Surgeries OR Surgeries, Laser Corneal OR Surgery, Laser Corneal OR Laser Corneal Surgery OR Keratectomy, Laser OR Keratectomies, Laser OR Laser Keratectomies OR Laser Keratectomy OR Photokeratectomy OR Photokeratectomies OR Laser Subepithelial Keratomileusis OR Subepithelial Keratomileusis, Laser OR LASEK OR Laser-Assisted Sub-Epithelial Keratomileusis OR Sub-Epithelial Keratomileusis, Laser-Assisted OR Keratomileusis, Subepithelial, Laser-Assisted OR Subepithelial Keratomileusis, Laser-Assisted OR Laser-Assisted Subepithelial Keratomileusis OR Subepithelial Keratomileusis, Laser Assisted OR Subepithelial Photorefractive Keratectomy OR Keratectomies, Subepithelial Photorefractive OR Keratectomy, Subepithelial Photorefractive OR Photorefractive Keratectomies, Subepithelial OR Photorefractive Keratectomy, Subepithelial OR Subepithelial Photorefractive Keratectomies OR Laser-Assisted Subepithelial Keratectomy OR Laser Assisted Subepithelial Keratectomy OR Laser-Assisted Subepithelial Keratectomies OR Subepithelial Keratectomies, Laser-Assisted OR Subepithelial Keratectomy, Laser-Assisted OR Subepithelial Keratectomy, Laser Assisted OR Laser-Assisted Stromal In Situ Keratomileusis OR Laser Assisted Stromal In Situ Keratomileusis OR Laser Intrastromal Keratomileusis OR Intrastromal Keratomileuses, Laser OR Intrastromal Keratomileusis, Laser OR Laser Intrastromal Keratomileuses OR Laser In Situ Keratomileusis OR LASIK OR Keratectomies, Photorefractive OR Keratectomy, Photorefractive OR Photorefractive Keratectomies  S2 Capsule Opacification OR Capsule Opacifications OR Opacification, Capsule OR Opacifications, Capsule OR Secondary Cataract OR Cataract, Secondary OR Cataracts, Secondary OR Secondary Cataracts OR cataract OR Lens Opacities OR Lens Opacity OR Opacities, Lens OR Opacity, Lens OR Cataract, Membranous OR Cataracts, Membranous OR Membranous Cataract OR Membranous Cataracts OR Pseudoaphakia OR Pseudoaphakias OR Capsule Opacification OR Capsule Opacifications OR Opacification, Capsule OR Opacifications, Capsule OR Secondary Cataract OR Cataract, Secondary OR Cataracts, Secondary OR Secondary Cataracts OR cataract OR Lens Opacities OR Lens Opacity OR Opacities, Lens OR Opacity, Lens OR Cataract, Membranous OR Cataracts, Membranous OR Membranous Cataract OR Membranous Cataracts OR Pseudoaphakia OR Pseudoaphakias  S3 Phakic Intraocular Lenses OR Intraocular Lenses, Phakic OR Lenses, Phakic Intraocular OR Lens, Intraocular, Phakic OR Phakic Intraocular Lens OR Intraocular Lens, Phakic OR Lens, Phakic Intraocular OR Multifocal Intraocular Lens OR Intraocular Lens, Multifocal OR Multifocal IOLs OR IOL, Multifocal OR Multifocal IOL OR Accommodating Intraocular Lenses OR Intraocular Lenses, Accommodating OR Accommodating Intraocular Lens OR Intraocular Lens, Accommodating OR Accommodating IOLs OR Accommodating IOL OR IOL, Accommodating OR IOLs, Accommodating OR Lenses, Intraocular OR Intraocular Lenses OR Lens, Intraocular OR Intraocular Lens OR Implantable Contact Lens OR Contact Lens, Implantable OR Lens, Implantable Contact  S4 clinical study OR Clinical Conference OR comparative study OR Consensus Development Conference OR Evaluation Study OR Meta-Analysis OR Multicenter Study OR Scientific Integrity Review OR Systematic Review OR Twin Study OR Validation Study OR Case Reports OR Clinical Trial OR Clinical Trial Protocol OR Clinical Trial, Veterinary OR Observational Study OR Observational Study, Veterinary OR Randomized Controlled Trial, Veterinary OR Adaptive Clinical Trial OR Clinical Trial, Phase I OR Clinical Trial, Phase II OR Clinical Trial, Phase III OR Clinical Trial, Phase IV OR Controlled Clinical Trial OR Randomized Controlled Trial  S5 S1 AND S2 AND S3 AND S4 | 63 |

**Supplemental Table S2.** Main characteristics of the included studies (as reported).

| Study | Country | patients, n | eyes, n | Type of IOLs | Refractive surgery procedures (numbers of eyes) | Sex  (male/female, n/n) | Age  (y)± SD | Years after refractive surgery | Mean preoperative  SE (D) ± SD | Mean axial length  (mm) ± SD | Mean keratometry (D) ± SD | Mean power implanted IOL (D) ± SD |
| --- | --- | --- | --- | --- | --- | --- | --- | --- | --- | --- | --- | --- |
| Christopher KL 2020^11^ | USA | 15 | 28 | EDOF IOLs | LASIK | 3/12 | 66.30 ± 7.90 | NA | NA | NA | NA | NA |
| Li QM 2020^12^ | China | 16 | 21 | Multifocal IOLs | 14 LASIK 7 PRK | 5/11 | 48.50 ± 8.90 | 13.20 ± 4.30 | -5.49 ± 5.75 | 27.70 ± 2.20 | 38.36 ± 2.27 | 17.64 ± 3.87 |
| Brenner LF 2019^13^ | Austria | 143 | 155/241 | Multifocal IOLs | Myopic | NA | 55.34 ± 4.84 | NA | -0.06 ± 0.78 | 24.88 ± 0.92 | 41.09 ± 1.82 | 21.29 ± 1.62 |
|  |  |  | 86/241 |  | Hyperopic | NA | 57.50 ± 7.22 | NA | 1.05 ± 0.90 | 22.93 ± 0.96 | 44.08 ± 1.81 | 22.38 ± 2.36 |
| Chow SW 2019^14^ | China | 13 | 20 | Multifocal IOLs | myopic LASIK | 2/11 | 53.00 ± 6.09 | within 20 years | -2.13 ± 2.20 | 26.5 ± 1.58 | NA | NA |
| Fisher B 2018^15^ | USA | 15/31 | 21/44 | Monofocal IOLs | Myopic/hyperopic LASIK 10/11 | 7/14 | 65.90 ± 6.90 | 12.50 ± 3.80 | -0.50 ± 1.67 | NA | 43.51 ± 3.11 | 20.5 ± 1.90 |
|  |  | 16/31 | 23/44 | Multifocal IOLs | Myopic/hyperopic LASIK 16/7 | 12/11 | 58.80 ± 6.20 | 12.10 ± 4.00 | -0.68 ± 1.49 | NA | 43.33 ± 2.7 | 20.1 ± 1.60 |
| Páez GF 2018^16^ | Spain | 17 | 30 | Multifocal IOLs | 16 hyperopic  14 myopic | 9/8 | 51.50 ± 6.30 | NA | -0.02 ± 2.30 | NA | 42 ± 2.8D | 21.00 ± 3.50 |
| Palomino-Bautista C 2018^17^ | Spain | 43 | 71 | EDOF IOLs | LASIK | 12/31 | NA | NA | -0.81 ± 0.13 | 25.17 ± 1.99 |  | 22.38 ± 2.36 |
| Vrijman V 2018^18^ | Netherlands | 40 | 40 | Multifocal IOLs | hyperopic LASIK | NA | 62.90 ± 6.90 | NA | 0.66 ± 1.13 | 23.06 ± 0.98 | 44.76 ± 1.59 | 21.11 ± 2.91 |
| Chang SM 2017^19^ | China | 23 | 27 | Multifocal IOLs | LASIK | 3/20 | 54.60 ± 4.60 | 10.20 ± 3.44 | -0.26 ± 1.31 | 25.59 ± 1.24 | NA | 20.89 ± 3.99 |
| Ferreira TB 2017^20^ | Portugal | 22 | 44 | Monofocal IOLs | Myopic LASIK | NA | 65.00 ± 5.70 | NA | OD -0.78 ± 0.85 OS -1.09 ± 0.92 | NA | NA | 20.00 ± 1.40 |
|  |  | 22 | 44 | EDOF IOLs |  |  | 62.20 ± 5.00 |  | OD -1.03 ± 0.99 OS -0.96 ± 0.88 |  |  | 20.20 ± 1.40 |
| Vrijman V 2017^21^ | Netherlands | 43 | 77 | Multifocal IOLs | Myopic LASIK 59/77  Myopic LASEK 6/77 Myopic PRK 12/77 | 20/23 | 58.90 ± 5.60 | NA | -0.38 ± 1.33 | 25.34 ± 1.28 | 40.66 ± 1.82 | 20.70 ± 2.46 |
| Muftuoglu O 2010^22^ | USA | 38 | 49 | Multifocal IOLs | Myopic LASIK | 17/21 | 57.00 ± 8.20 | 5.80 ± 2.50 | -0.82 ± 1.65 | 25.06 ± 1.83 | 39.62 ± 2.02 | 19.86 ± 2.90 |
| Fernández-Vega L 2009^10^ | Spain | 46 | 80 | Spherical Multifocal IOLs | Myopic LASIK | 4/8 | 53.60 ± 5.23 | NA | -0.06 ± 0.33 | 25.60 ± 0.90 | 39.49 ± 1.8 | NA |
|  |  | 12 | 22 | Aspheric Multifocal IOLs |  | 6/11 | 55.04 ± 6.06 |  | -0.02 ± 0.25 | 25.78 ± 1.84 | 39.71 ± 2.57 |  |
|  |  | 16 | 32 | Control |  | 6/10 | 52.37 ± 4.06 |  | 0.05 ± 0.33 | 25.29 ± 0.90 | 39.86 ± 1.73 |  |

IOL, intraocular lense; MIOLs, multifocal intraocular lenses; EDOF, extended depth-of-focus; LASIK, laser in-situ keratomileusis; PRK, photorefractive keratectomy; SE, spherical equivalent; NA, not available.

**Supplemental Table S3.** Quality assessment of 13 studies by Methodological Index for Non-randomized studies (MINORS).

| Study | Study design | Level of evidenvce | A clearly stated aim | Inclusion of consecutive patients | Prospective collection of data | Endpoints appropriate to the aim of the study | Unbiased assessment of the study endpoint | Follow-up period appropriate to the aim of the study | Loss to follow up less than 5% | Prospective calculation of the study size | An adequate control group | Contemporary groups | Baseline equivalence of groups | Adequate statistical analyses | Quality assessment |
| --- | --- | --- | --- | --- | --- | --- | --- | --- | --- | --- | --- | --- | --- | --- | --- |
| Christopher KL 2020^11^ | retrospective review | IV | 2 | 2 | 2 | 2 | 0 | 0 | 2 | 0 | / | / | / | / | 10/16 |
| Li QM 2020^12^ | retrospective case ceries | IV | 2 | 2 | 2 | 2 | 0 | 2 | 2 | 0 | / | / | / | / | 12/16 |
| Brenner LF 2019^13^ | retrospective case ceries | IV | 2 | 2 | 2 | 2 | 0 | 2 | 2 | 0 | / | / | / | / | 12/16 |
| Chow SW 2019^14^ | retrospective case ceries | IV | 2 | 2 | 2 | 2 | 0 | 2 | 2 | 0 | / | / | / | / | 12/16 |
| Fisher B 2018^15^ | retrospective review | IV | 2 | 2 | 2 | 2 | 0 | 2 | 2 | 2 | / | / | / | / | 14/16 |
| Páez GF 2018^16^ | retrospective study | IV | 2 | 2 | 2 | 2 | 0 | 2 | 2 | 0 | / | / | / | / | 12/16 |
| Palomino-Bautista C 2018^17^ | prospective study | IV | 2 | 2 | 2 | 2 | 0 | 2 | 2 | 0 | / | / | / | / | 12/16 |
| Vrijman V 2018^18^ | retrospective study | IV | 2 | 2 | 2 | 2 | 0 | 2 | 2 | 0 | / | / | / | / | 12/16 |
| Chang SM 2017^19^ | prospective case ceries | IV | 2 | 2 | 2 | 2 | 0 | 2 | 2 | 0 | / | / | / | / | 12/16 |
| Ferreira TB 2017^20^ | prospective comparative nonrandomized study | III | 2 | 2 | 2 | 2 | 0 | 2 | 2 | 0 | 2 | 2 | 2 | 2 | 20/24 |
| Vrijman V 2017^21^ | retrospective study | IV | 2 | 2 | 2 | 2 | 0 | 2 | 2 | 0 | / | / | / | / | 12/16 |
| Muftuoglu O 2010^22^ | retrospective comparative case serie | IV | 2 | 2 | 2 | 2 | 0 | 2 | 2 | 0 | 2 | 2 | 1 | 2 | 19/24 |
| Fernández-Vega L 2009^10^ | prospective comparative nonrandomized study | III | 2 | 2 | 2 | 2 | 0 | 2 | 2 | 0 | 2 | 2 | 2 | 2 | 20/24 |

**Supplemental Table S4.** Clinical outcomes of presbyopia-correcting IOLs implantation in cataract surgery after laser refractive surgery.

| Study | Follow-up time (months) | Mean preoperative  SE (D) ± SD | Mean postoperative SE (D) ± SD | postoperative SE within  ± 0.50 D | postoperative SE within  ± 1.00 D | UDVA | CDVA | UIVA | UNVA | spectacles independence |
| --- | --- | --- | --- | --- | --- | --- | --- | --- | --- | --- |
| Christopher KL 2020^11^ | NA | NA | -0.14 ± 0.45 | 78.57% | 96.43% | ≥1.0 8/28 (28.57%) ≥0.8 24/28 (85.71%) | ≥1.0 20/28 (71.4%) ≥0.8 26/28 (92.9%) | NA | NA | NA |
| Li QM 2020^12^ | 3 | -5.49 ± 5.75 | -0.56 ± 0.49 | 47.62% | 90.48% | 0.02 ± 0.07 logMAR ≥1.0 9/21 (42.86%) ≥0.8 16/21 (76.19%) | 0.00 ± 0.05 logMAR ≥1.0 18/21 (85.71%) ≥0.8 21/21 (100%) | 0.10 ± 0.10 logMAR | 0.15 ± 0.11 logMAR | Far distance 16/16 (100%)  Intermediate distance 16/16 (100%)  Near distance13/16 (81.25%) |
| Brenner LF 2019^13^ | 6 | -0.06 ± 0.78 | -0.25 ± 0.38 | 80.00% | 97,4% | 0.88± 0.20 (Snellen) | 1.06± 0.10 (Snellen) | NA | 5.11 ± 0.46 (point type) | NA |
|  |  | 1.05 ± 0.90 | -0.02 ± 0.42 | 82.60% | 98.80% | 0.85± 0.19 (Snellen) | 1.03 ± 0.10 (Snellen) | NA | 5.25 ± 0.75 (point type) |  |
|  |  |  |  | 80.90% | 97.90% | ≥1.0 112/241 (46.47%) ≥0.8 196/241 (81.33%) | NA | NA | NA |  |
| Chow SW 2019^14^ | 6 | -2.13 ± 2.20 | -0.92 ± 0.76 | 55.00% | NA | 0.28 ± 0.29 logMAR | 0.06 ± 0.14 logMAR | NA | 0.02 ± 0.05 logMAR | NA |
| Fisher B 2018^15^ | 3 | -0.50 ± 1.67 | NA | NA | NA | ≥1.0 6/21 (28.57%) ≥0.8 14/21 (66.67%) | NA | NA | NA | NA |
|  |  | -0.68 ± 1.49 | NA |  |  | ≥1.0 10/23(43.47%) ≥0.8 18/23(78.26%) |  |  |  |  |
| Páez GF 2018^16^ | 12 | -0.02 ± 2.3 | -0.09 ± 0.30 | NA | NA | 0.90± 0.1 (decimal scale) | 0.95± 0.1(decimal scale) | NA | -1.00± 0.02 (decimal scale) | Far distance 17/17 (100%)  Intermediate distance 17/17 (100%)  Near distance14/17 (82.35%) |
| Palomino-Bautista C 2018^17^ | 3 | -0.81 ± 0.13 | -0.46 ± 0.55 | 61.97% | 85.92% | NA | 0.05 ± 0.12 logMAR | NA | 0.04 ± 0.09 logMAR | NA |
| Vrijman V 2018^18^ | 3 | 0.66 ± 1.13 | 0.04 ± 0.92 | 62.50% | 87.50% | 0.16 ± 0.18 logMAR | 0.01 ± 0.08 logMAR | NA |  | NA |
| Chang SM 2017^19^ | 15 | -0.26 ± 1.31 | -0.27 ± 0.83 | 63.00% | 89.00% | 0.13 ± 0.15 logMAR ≥1.0 8/27 (29.63%) ≥0.8 18/27 (66.67%) | -0.03 ± 0.07 logMAR ≥1.0 25/27 (92.59%) ≥0.8 27/27 (100%) | 0.22 ± 0.15 logMAR ≥1.0 8/23 (34.78%) ≥0.8 14/23 (60.87%) | 0.16 ± 0.15 logMAR ≥1.0 2/23 (8.70%) ≥0.8 8/23 (34.78%) | Far distance 22/23 (95.65%)  Intermediate distance 20/23 (86.96%)  Near distance 20/23 (86.96%) |
| Ferreira TB 2017^20^ | 4 | OD -0.78 ± 0.85 OS -1.09 ± 0.92 | OD -0.20 ± 0.78 OS -0.31 ± 0.75 | 56.82% | 86.36% | OD 0.04 ± 0.10 logMAR OS 0.01± 0.08 logMAR ≥1.0 24/44 (54.50%) ≥0.8 42/44 (95.45%) | OD -0.06 ± 0.06 logMAR OS -0.10 ± 0.06 logMAR | ≥1.0 3/22 (13.60%) ≥0.8 12/22 (54.55%) | ≥1.0 0/22 (0%) ≥0.8 0/22 (0%) | 0 |
|  |  | OD -1.03 ± 0.99 OS -0.96 ± 0.88 | OD -0.27 ± 0.68 OS -0.43 ± 0.70 | 55.00% | 59.09% | OD 0.03 ± 0.07 logMAR OS 0.04 ± 0.09 logMAR ≥1.0 24/44 (52.50%) ≥0.8 42/44 (95.45%) | OD -0.05 ± 0.05 logMAR OS -0.04 ± 0.08 logMAR | ≥1.0 22/22 (100%) ≥0.8 22/22 (100%) | ≥1.0 8/22 (18.2%) ≥0.8 26/22 (59.09%) | Far distance 21/22 (95.45%)  Intermediate distance 22/22 (100%)  Near distance 10/22 (45.45%) |
| Vrijman V 2017^21^ | 3 | -0.38 ± 1.33 | -0.38 ± 0.78 | 57.14% | 85.71% | 0.14 ± 0.22 logMAR | NA | NA | 0.10 ± 0.10 logMAR | NA |
| Muftuoglu O 2010^22^ | 6 | -0.82 ± 1.65 | NA | 69.39% | 81.63% | ≥0.8 36/49 (73.47%) | NA | NA | ≥J1 36/49(73.47%) | NA |
| Fernández-Vega L 2009^10^ | 6 | -0.06 ± 0.33 | -0.15 ± 0.38 | NA | NA | 0.086 ± 0.116 logMAR | 0.035 ± 0.06 logMAR | NA | NA | NA |
|  |  | -0.02 ± 0.25 | -0.05 ± 0.27 | NA | NA | 0.041 ± 0.121 logMAR | 0.004 ± 0.058 logMAR | NA | NA |  |
|  |  | 0.05 ± 0.33 | NA | NA | NA | 0.086 ± 0.116 logMAR | 0.019 ± 0.036 logMAR | NA | NA |  |

SE, spherical equivalent; UDVA, uncorrected distance visual acuity; CDVA, corrected distance visual acuity; UIVA, uncorrected intermediate visual acuity; UNVA, uncorrected near visual acuity; NA, not available.
