# Supplementary material for: Presbyopia-Correcting Intraocular Lenses Implantation in Eyes After Corneal Refractive Laser Surgery: A Meta-Analysis and Systematic Review
Source: Front Med (Lausanne). 2022 Apr 11;9:834805. doi: 10.3389/fmed.2022.834805 (PMC9035540; doi:10.3389/fmed.2022.834805)
Supplement: Supplementary file 2 [file Data_Sheet_1.docx]

**Supplemental figure legends:**

**Supplemental Figure S1**. Forest plot of uncorrected distance visual acuity (UDVA) after splitting diffractive MIOLs into bifocal and trifocal IOLs. The proportion of the eyes with a postoperative visual acuity ≥ 20/25 was represented in this graph.

**Supplemental Figure S2**. Forest plot of spectacle independence of far, intermediate and near distance after excluding the study of Ferreira et al.

**Supplemental Figure S3**. Forest plot of photic disturbance of halos and glare after removing the study by Chang et al.

**Supplemental Figure S4**. Forest plot of postoperative refraction after removing the study by Brenner et al. The proportion of eyes with a postoperative refraction of 0.5 diopters (A) and 1.0 diopters (B) from the target refraction according to follow-up time (< 6 months versus ≥ 6 months).

**Supplemental Figure S5**. Forest plot of postoperative refraction after removing study by Brenner et al. The proportion of eyes with a postoperative refraction of 0.5 diopters from the target refraction according to mean axial length (< 26mm versus ≥ 26mm).
