# Supplementary figures and images for: Presbyopia-Correcting Intraocular Lenses Implantation in Eyes After Corneal Refractive Laser Surgery: A Meta-Analysis and Systematic Review
Source: Front Med (Lausanne). 2022 Apr 11;9:834805. doi: 10.3389/fmed.2022.834805 (PMC9035540; doi:10.3389/fmed.2022.834805)

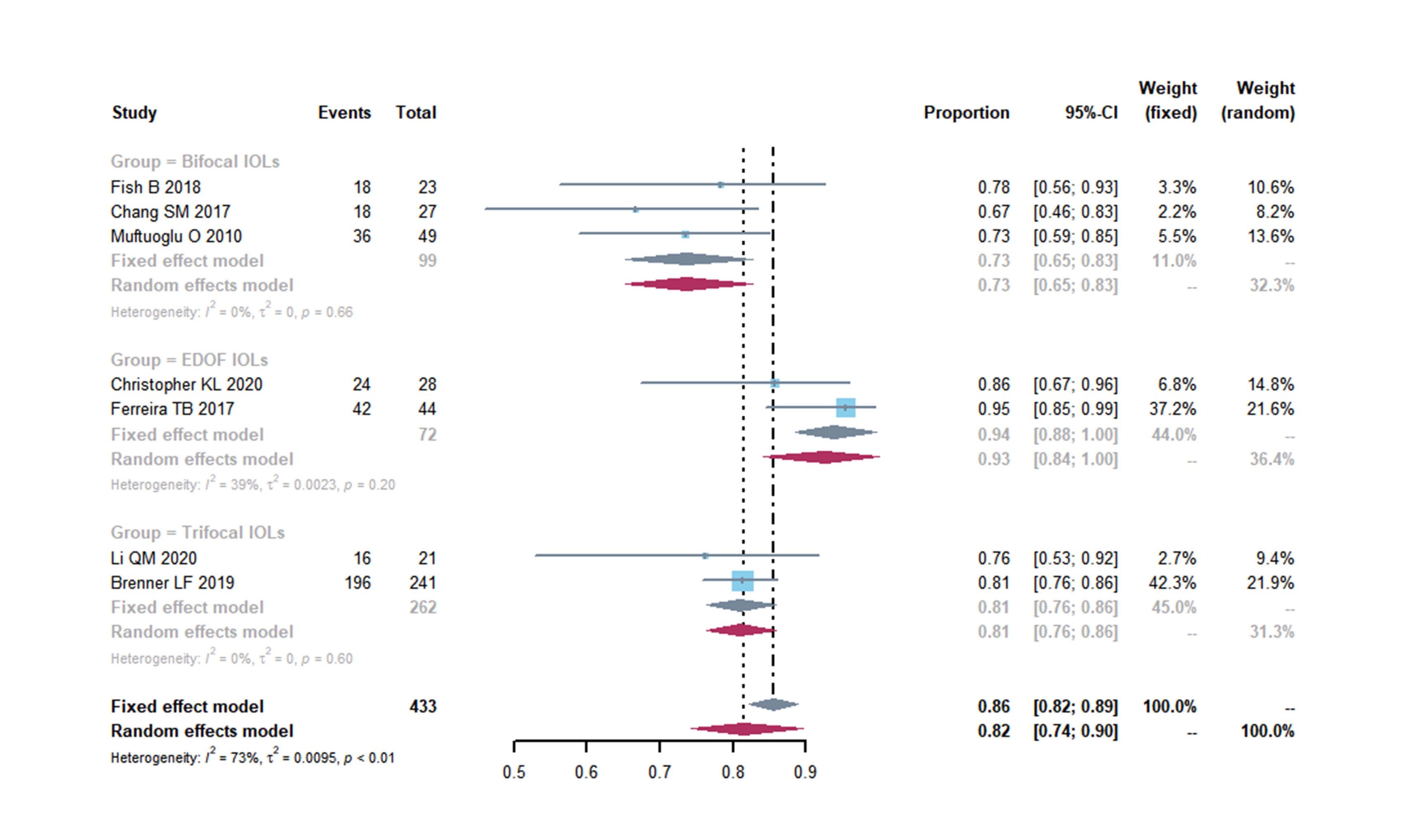

Supplement: Supplementary file 3 [file Image_1.tiff]

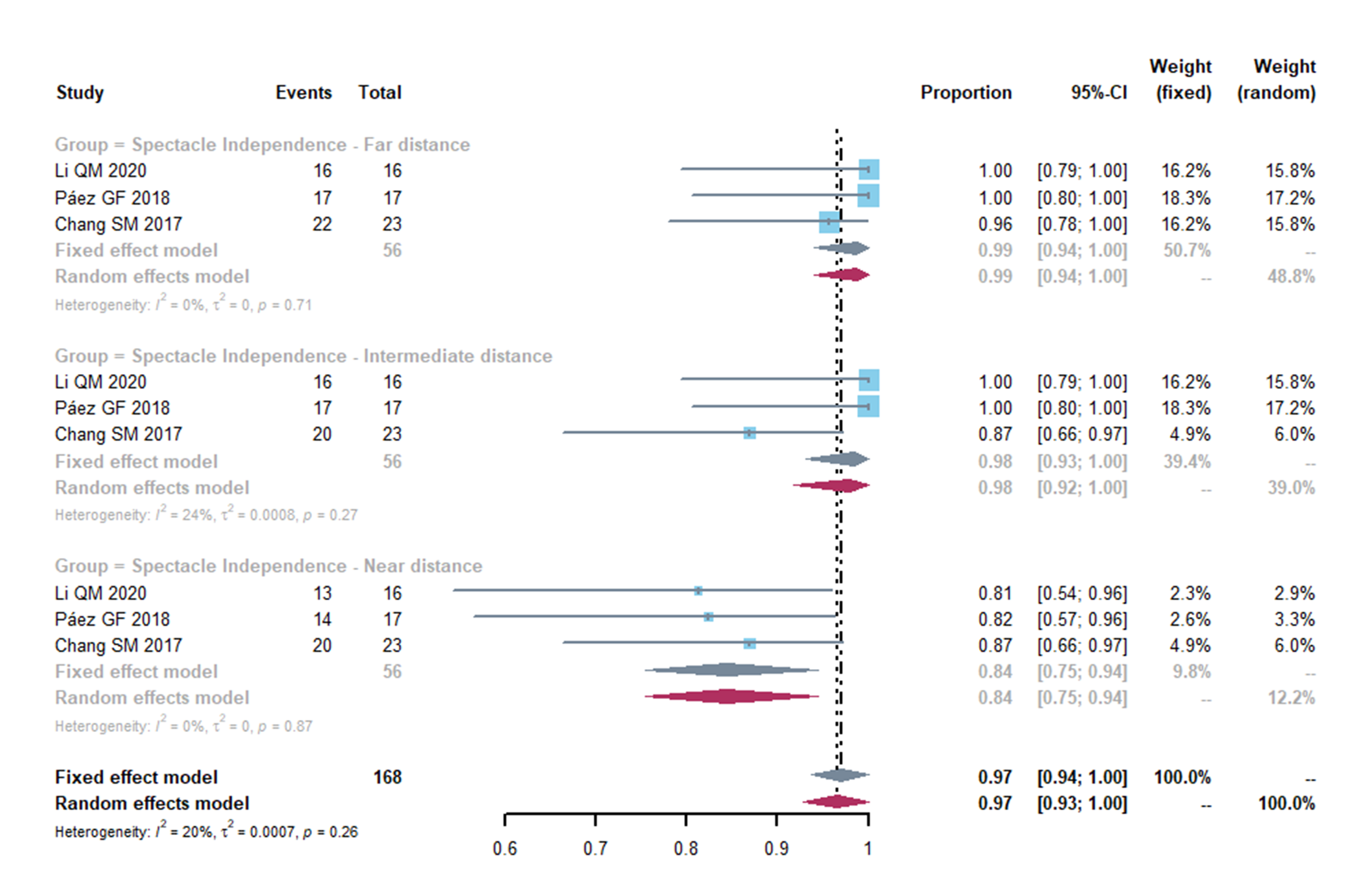

Supplement: Supplementary file 4 [file Image_2.tiff]

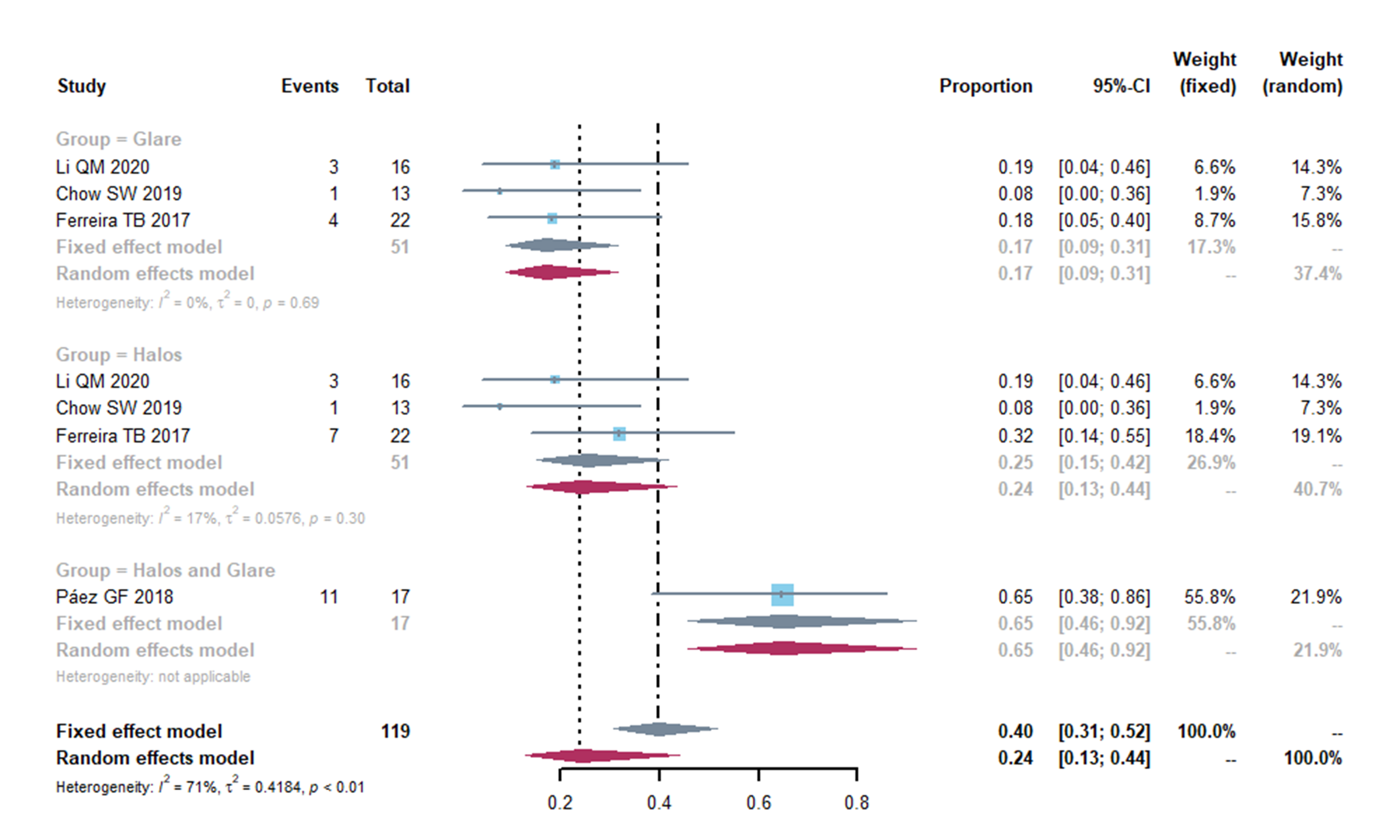

Supplement: Supplementary file 5 [file Image_3.tiff]

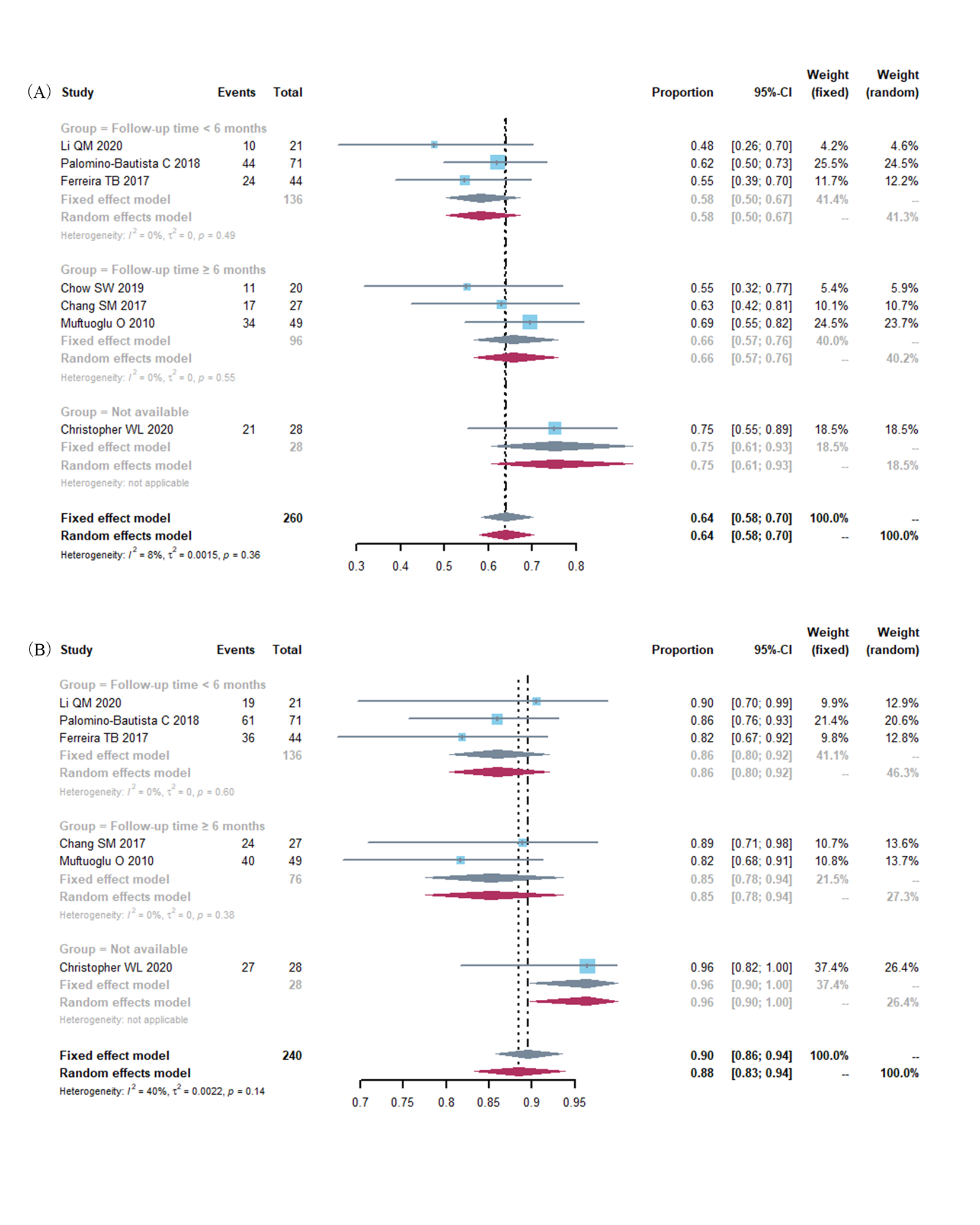

Supplement: Supplementary file 6 [file Image_4.tif]

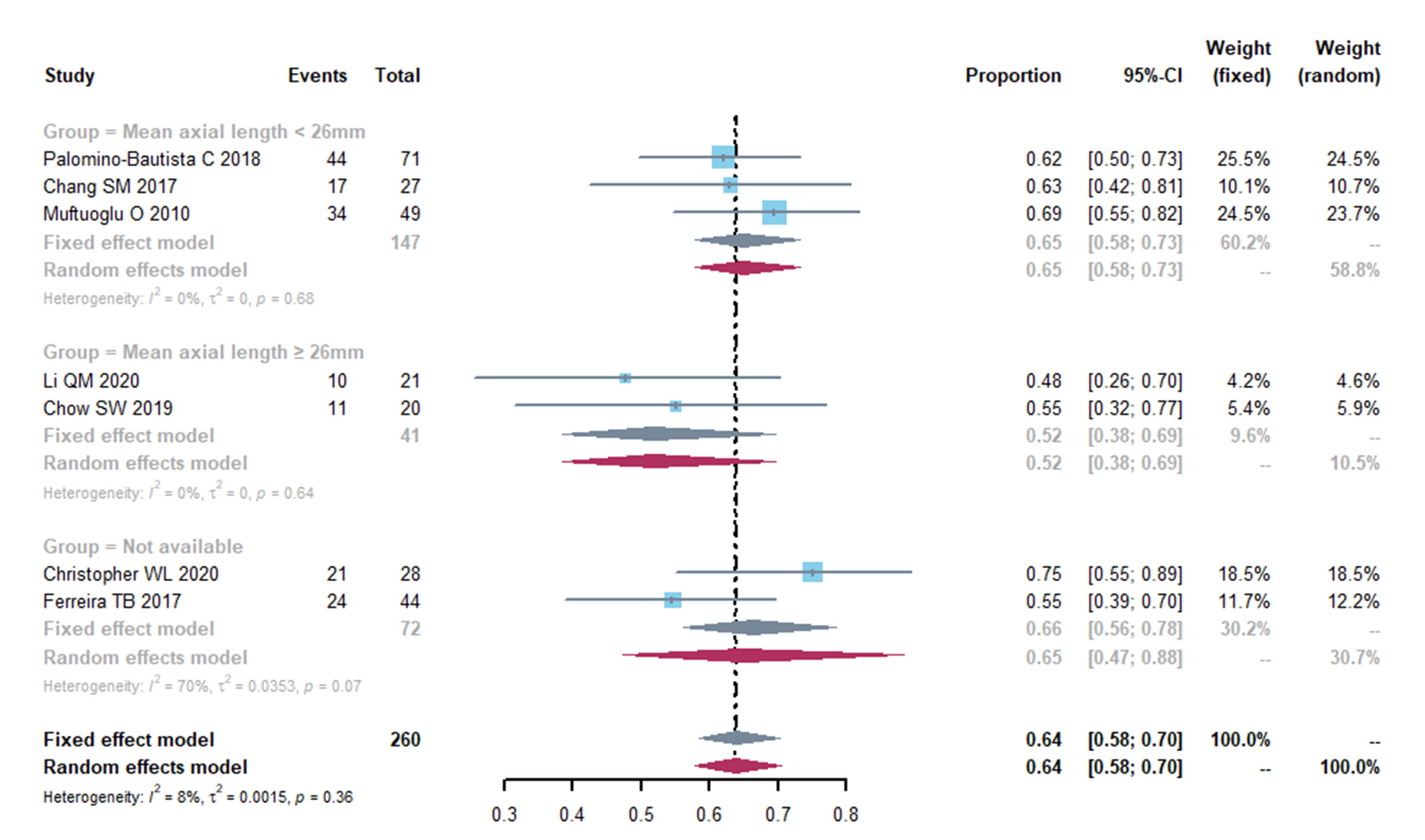

Supplement: Supplementary file 7 [file Image_5.tiff]
